# A virtual patient simulation modeling the neural and perceptual effects of human visual cortical stimulation, from pulse trains to percepts

**DOI:** 10.1038/s41598-024-65337-1

**Published:** 2024-07-29

**Authors:** Ione Fine, Geoffrey M. Boynton

**Affiliations:** 1 Department of Psychology, University of Washington, Seattle, 98195 USA; 2 Faculty of Biological Sciences, University of Leeds, Leeds, UK

**Keywords:** Cortical prosthesis, Electrical stimulation, Bionic eye, Sight restoration, Blindness, Sight recovery, Computational neuroscience, Visual system, Human behaviour

## Abstract

The field of cortical sight restoration prostheses is making rapid progress with three clinical trials of visual cortical prostheses underway. However, as yet, we have only limited insight into the perceptual experiences produced by these implants. Here we describe a computational model or ‘virtual patient’, based on the neurophysiological architecture of V1, which successfully predicts the perceptual experience of participants across a wide range of previously published human cortical stimulation studies describing the location, size, brightness and spatiotemporal shape of electrically induced percepts in humans. Our simulations suggest that, in the foreseeable future the perceptual quality of cortical prosthetic devices is likely to be limited by the neurophysiological organization of visual cortex, rather than engineering constraints.

## Introduction

A variety of sight recovery technologies are now in development worldwide^1^. At least eight groups are developing retinal electronic implants, with two devices approved for patients^2–9^ and others in clinical trials^10^. Optogenetics^11,12^ is another promising direction of research, with preliminary results from one clinical trial reporting limited restored vision^13^, and two other clinical trials in early stages^14,15^. Genetic treatment for Leber congenital amaurosis is clinically approved^16^ with many other genetic treatments in development^17^. Retinal epithelium^18,19^ and stem-cell^20^ transplants are making rapid progress with several Phase I/II clinical trials underway, and a wide variety of other promising therapies are under development^21–23^.

However, all of these are retinal interventions, and cannot be used to treat diseases such as retinal detachment or pediatric congenital glaucoma that result in irreparable damage to the ganglion cells of the retina or the optic nerve. This has motivated interest in cortical sight recovery technologies. Since 2017 three clinical trials of visual cortical prostheses have begun, one with surface electrodes (second sight medical products, Orion^24,25^, and the other two using depth electrodes^26,27^).

These clinical trials rest upon a longstanding and substantial body of literature examining the effects of both acute and chronic cortical stimulation, see Table 1. However, to date, results from this wide collection of studies have been almost entirely descriptive. Here, for the first time, we show that a great deal of the literature on human electrical stimulation of early visual cortex can be modeled using a simple model based on the neurophysiological architecture of V1.

**Table 1:** A subset of the papers describing the perceptual effects of cortical electrical stimulation in humans.

Date	Paper	Electrodes/protocol	Experiments
1968	Brindley & Lewin (1968)^28^	Acute surface electrodes	Pulse duration vs. threshold; frequency vs. threshold; the relationship between cortical electrode location and phosphene location
1974	Dobelle & Mladejovsky (1974)^29^	Acute surface electrodes	Pulse duration vs. threshold; frequency vs. threshold; train duration vs. amplitude; the relationship between cortical electrode location and phosphene location
1977	Rushton & Brindley (1977)^30^	Chronic surface electrodes	The stability of the location of phosphenes; the relationship between cortical electrode location and phosphene location
1979	Evans et al. (1979)^31^	Chronic surface electrodes	Brightness vs. amplitude; brightness vs. frequency; brightness vs. train duration; the relationship between cortical electrode location and phosphene Location
1979	Girvin et al. (1979)^32^	Chronic surface electrodes	Pulse duration vs. threshold; frequency vs. threshold; train duration vs. threshold; pulse spacing and number vs. threshold
1979	Dobelle et al. (1979^33^	Chronic surface electrodes	Pulse duration vs. threshold; pulse timing vs. threshold; brightness vs. amplitude; the relationship between cortical electrode location and phosphene location
1996	Schmidt et al. (1996)^34^	Acute depth electrodes	Thresholds for anodic/cathodic first stimulation; desensitization with repeated stimulation;
2016	Winawer & Parvizi (2016)^35^	Acute surface electrodes	Size vs. amplitude; the relationship between cortical electrode location and phosphene location and size
2017	Bosking et al. (2017)^24^	Acute surface electrodes	Size vs. amplitude; the relationship between cortical electrode location and phosphene location and size
2020	Beauchamp et al. (2020)^25^	Chronic surface electrodes	Form perception during stimulation of multiple electrodes, simultaneously or sequentially
2021	Fernández et al. (2021)^27^	Semi-chronic depth electrodes	Threshold and brightness vs. amplitude, form perception during stimulation of multiple electrodes
2021	Oswalt et al. (2021)^36^	Chronic surface electrodes	Stability of phosphene location, retinotopic location
2022	Bosking et al. (2022)^37^	Chronic surface electrodes	Location and size of phosphenes as a result of simultaneous vs. single electrode stimulation
2022	Salas et al. (2022)^38^	Chronic surface electrodes	Temporal interactions in sequential stimulation

Our model approximates human cortical magnification^39,40^, orientation preference^41^, ocular dominance^42,43^, receptive field size^44^, and the on- and off- structure of simple and complex neurons^45,46^, based on previous studies of V1 neuronal architecture. Our model of the temporal integration of current and the resulting conversion to neural signal strength is loosely based on our previous model of retinal prosthetic stimulation^47,48^. The percept resulting from the stimulation of these neuronal populations is based on a linear sum of each cells’ receptive field, weighted by the neural signal strength at that location at each moment in time. Despite its simplicity and lack of fitted parameters, our model successfully predicts a wide variety of cortical stimulation data.

Models like these can be considered to be ‘virtual patients’ and play a role analogous to that of virtual prototypes (also known as digital manufacturing). For researchers and companies, these models can guide the placement of existing devices, aid in new technology development, and provide quantitative tests of whether we have a full understanding of how cortical prosthesis technologies interface with the neurophysiological organization of visual cortex. For entities such as the FDA and Medicare, these models can provide insights into what sorts of visual tests/metrics will be important for evaluating devices. Finally, for surgeons and patient families, these models will provide more realistic expectations than current ‘scoreboard’ models that misleadingly assume that each electrode produces an equally sized circular phosphene, analogous to the lights on the scoreboard at a football game.

## Results

Written in Matlab, our model (https://github.com/VisCog/p2p-cortical) has a modular structure designed to make it easy to simulate novel implants and stimuli, thereby allowing us to simulate a wide range of data from the human (and primate) literature. Unless otherwise specified, all figures in the paper are based on simulation of the full model, using the parameters described in Table 2, with only *s*, the linear scaling of the perceptual response with current, varying across experiments.

**Table 2:** Major parameters used in the model.

	Electric field, K = 675 μA/mm^2^	Fall-off in current as a function of distance from the electrode for surface electrodes. K = 675 μA/mm^2^ was fixed based on neurophysiological literature examining current spread from an electrode with a distant ground^49^
Spatial model	Ocular dominance, orientation and on–off maps, Z = 0.5mm	The scale of the bandpass filter used to create orientation (θ), ocular dominance (w_od_), and on–off (${\text{w}}_{\text{on}/\text{off}}, {\updelta }_{\text{on}/\text{off}})$ maps. Fixed based on neurophysiological literature. Method based on Ref.^43^, similar to the modeling of Ref.^46^, size of filter based on Ref.^42^
	Receptive field size, long axis ${\upsigma }_{\text{rf}}=0.16+0.08\text{ x eccentricity}$	Receptive field size, long axis. Fixed based on single-cell macaque neurophysiological literature^44^
	Receptive field size, short axis ${\upsigma }_{\text{rf}}/4$	Receptive field size, short axis. Fixed based on single-cell neurophysiological literature^44,50^
	The relative strength of responses to stimulus increments and decrements $\text{cell response}={\text{w}}_{\text{on}/\text{off}}\cdot \text{ON}$ + ${\upomega \cdot (1-\text{w}}_{\text{on}/\text{off}})\cdot \text{OFF}$ $\upomega =0.8$	Although off cells are more numerous than on cells, and saturate more slowly as a function of contrast^51–53^ studies of electrical stimulation, for both retina and cortex, consistently find that dark phosphenes are only observed very near threshold^24,47^. We simulated this by weighting on-subunit responses more heavily than off responses, model performance was robust across a wide range of $\upomega$
	On/off subunit separation ${\updelta }_{\text{on}/\text{off}}$ = 2	Controls the distribution of separations between the on subunit (responds to increments) and the off subunit (responds to decrements). Fixed based on the neurophysiological literature^45^, see “STAR methods”
Temporal model	1st stage, ‘spiking response strength’ τ1 = 0.3ms	τ1, the first stage of current integration, based on a 1-stage leaky integrator, Fixed based on neurophysiological literature^54^
	Refractory period τ_r_ = 100 δ = 0.001s	τ_r_ and δ determine spiking response strength attenuation and timing. Fitted based on thresholds as a function of frequency^29,32^
	2nd stage τ_2_ = 25ms	Second slow integration stage based on a 3-stage leaky integrator. τ_2_ was fit based on data examining brightness as a function of frequency, pulse width and pulse train duration^35^
Response scaling & compression	${\text{s}}_{\text{in}}$ = 0.57 (default) ${\text{R}}_{\text{out}}={\text{s}}_{\text{out}}\text{ tanh}(\text{s }\times {\text{R}}_{\text{in}}/\text{p}$) ${\text{s}}_{\text{out}}$= 10 p = 15.6 ${\uptheta }_{\text{draw}}$= 1	Response scaling and compression parameters, ${\text{s}}_{\text{in}}$ represents a scaling of sensitivity of current and is likely driven by the factors such as the height of the electrode on the cortical surface and current density. ${\text{s}}_{\text{in}}$ was allowed to vary when fitting individual datasets. We used a default of 0.57 for simulations, based on Ref.^35^ The choice of ${\text{s}}_{\text{out}}$ = 10 was chosen to match experimental 1–10 brightness rating scales^24,35^ p was fit based on brightness rating data^35^ ${\uptheta }_{\text{draw}}$ was fixed at 1, the lowest brightness rating on the brightness scale

### Transformation from pulse trains to perceptual intensity over time

**Figure 1:**
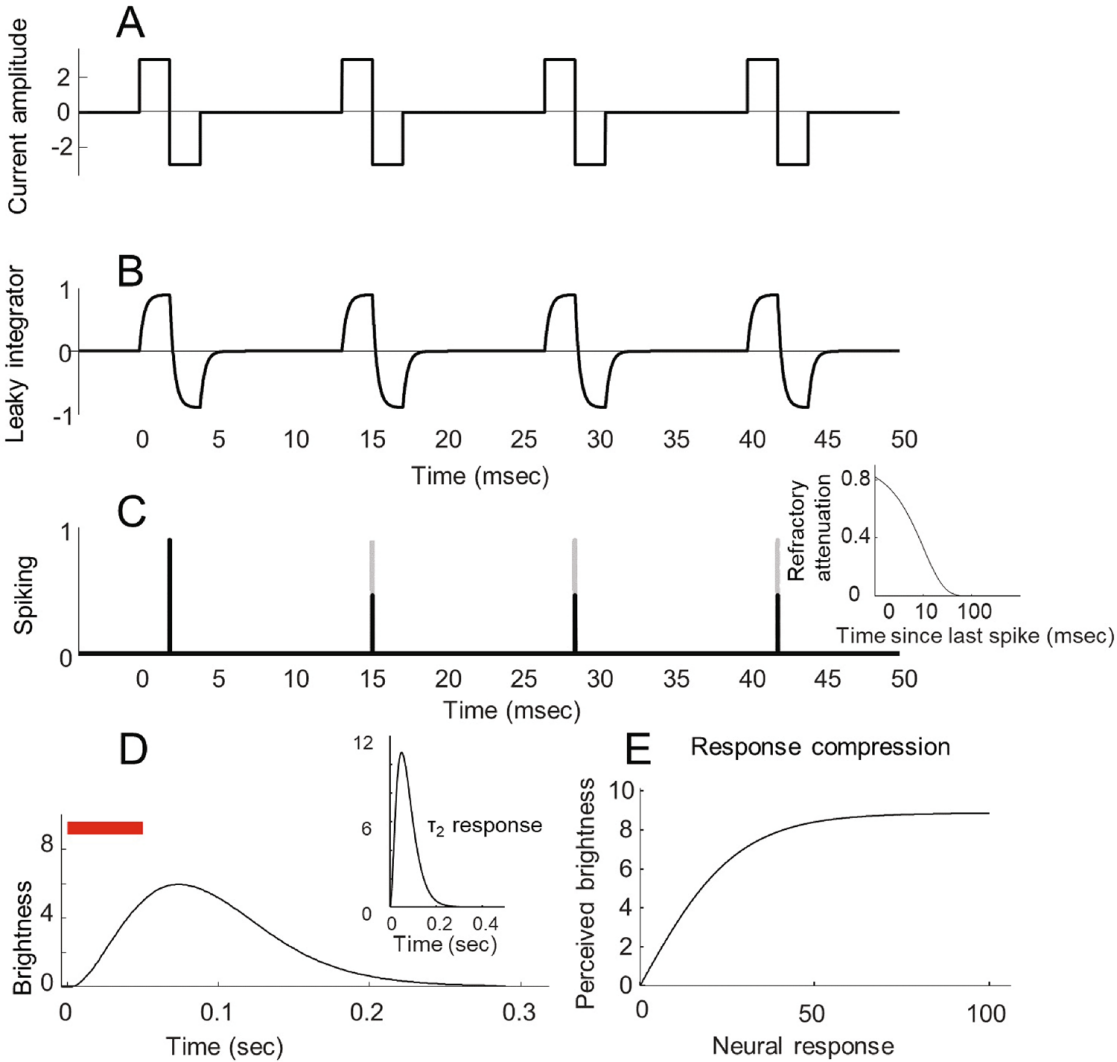
Schematic of the transformation from pulse trains to perceptual intensity over time. (**A**) Cathodic-first pulse train with a pulse width of 2 ms, frequency 75 Hz and pulse train duration of 50 ms. (**B**) The output of the first stage of temporal integration of current. (**C**) The peak of each leaky integrator response provides a measure of ‘spike response strength’. Gray and black solid lines show spike response strength before and after attenuation due to the refractory period. The inset shows the strength of refractory attenuation as a function of time since the previous burst of spiking activity. (**D**) Perceived brightness as a function of time for this pulse train. The final stages of the model include slow temporal integration (modeled by a 3-stage leaky integrator), followed by (**E**) a compressive response non-linearity.

A rapid temporal integration stage, long thought to reflect cellular integration of current^55,56^, was used to generate a measure of the ‘strength of spiking activity’. We further assumed a spiking refractory period, followed by a slower integration stage and a compressive nonlinearity, Fig. 1. The relationship between extracellular stimulation, neuronal depolarization and spiking thresholds has been modeled at various levels of complexity^57–59^. We used a simple well-established model which assumes that the resting state membrane potential change is proportional to the second-order spatial derivative of the extracellular potential over the cell^55,56^. This was modeled by a one-stage leaky integrator, for which the rate of change of depolarization is proportional to the current level of depolarization plus the input current^60–62^. At a single cell level, because electrical pulse durations are short compared to the refractory period, a neuron will (almost always) produce a single spike rather than multiple spikes. However, neurons vary in the sensitivity of their activating function. Consequently, as the current amplitude of a pulse increases, more and more cells under the electrode will reach their depolarization threshold. Thus, in our model, the output of the first stage, ‘spike response strength’ should not be thought of as representing spikes per se, but as reflecting the recruitment of spikes from a population of cells with activation functions that vary in sensitivity. Our compressive non-linearity captures saturation within this population of cells as well as the effects of more complex cortical gain control mechanisms.

### Transformation from visual space to the cortical surface

**Figure 2:**
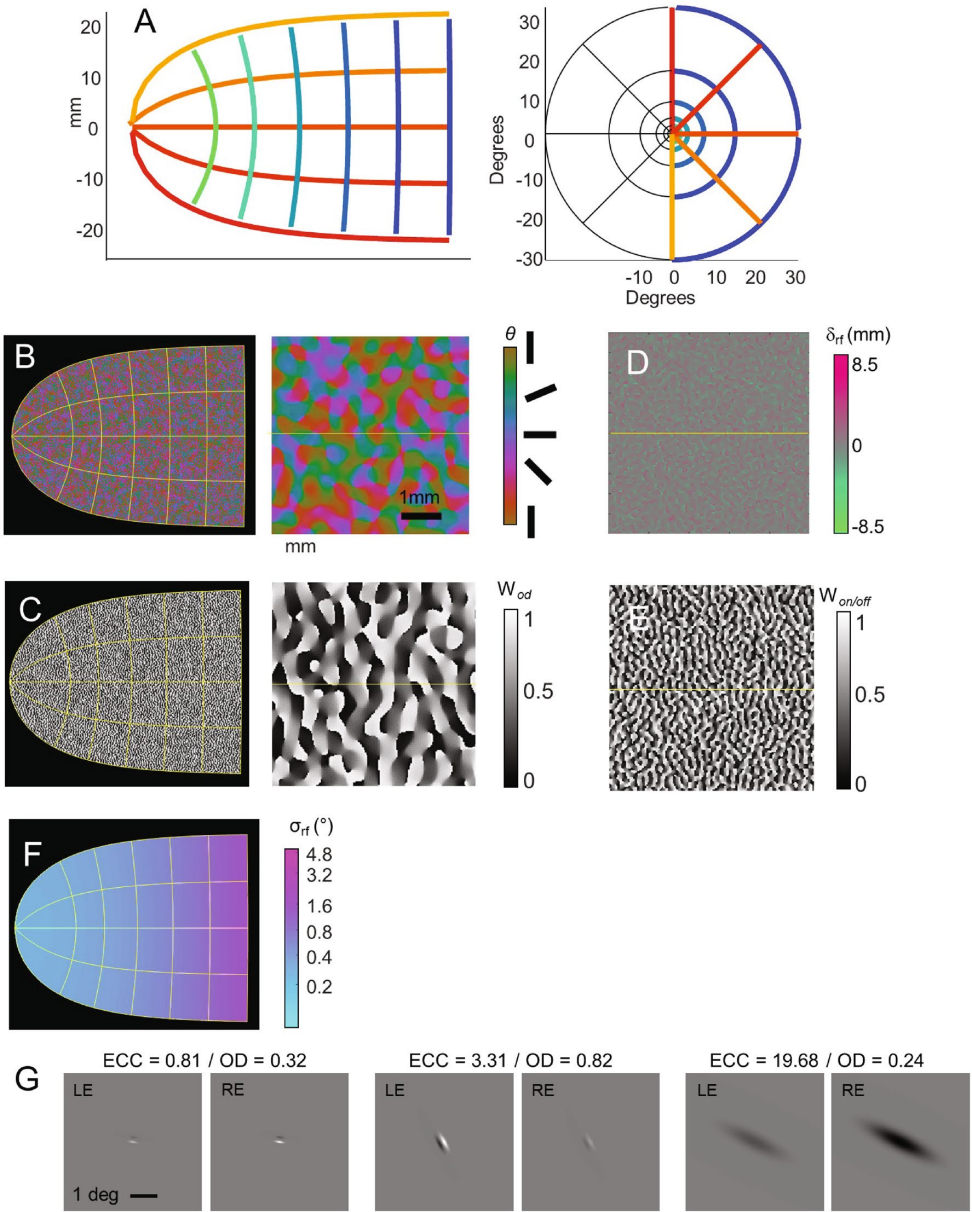
Schematic of our cortical model. (**A**) Transformation from visual space to the cortical surface, based on Ref.^63^. Simulated V1 maps on the human cortical surface for (**B**) orientation pinwheel maps (entire cortical map and a 5 mm^2^ region), (**C**) ocular dominance columns (entire cortical map and a 5mm^2^ region), where the neural response is described as W_od_ × LE + (1–W_od_ × RE) (**D**) on- vs. off-subunit spatial separation (5 mm^2^ region), (**E**) on vs off relative strength (5mm^2^ region), where the relative strength of the response to increments (OFF) and decrements (ON) is described as W_on/off_ × ON + (1–W_on/off_ × OFF), and (**F**) receptive field sizes (entire cortical map). (**G**) Example individual receptive fields in V1: each receptive field is shown centered in a 5° region of the visual field (*ECC* eccentricity, *OD* ocular dominance ratio, *LE/RE* left eye/right eye).

We used a template derived from a conformal map developed by Schwartz et al.^40,63,64^, in which two-dimensional visual space is projected onto the two-dimensional flattened cortex (*w* = *log(z* + *a)*, where *z* is a complex number representing visual space, *w* is the corresponding location in cortical space, and *a* = 0.5 deg), Fig. 2A. This transformation (in conjunction with the Benson et al. template that maps from human cortical anatomy to retinal location^65^) has previously been used successfully in the cortical stimulation literature to map the cortical location of electrodes to visual space^35,36^.

### Ocular dominance columns, orientation pinwheels and receptive fields

Figure 2B–G, were simulated based on Rojer and Schwartz^43^. Orientation columns (*θ*, Panel B) were modeled by bandpass filtering white noise in the complex domain, with the resulting angle representing orientation preference (the scale of the bandpass filter was based on Ref.^42^). We then extended the model to include ocular dominance columns (*w*_*od*_, Panel C) as the gradient of the same filtered white noise along a single direction, thereby generating orthogonal ocular dominance and orientation columns that closely resemble measured ocular dominance and orientation pinwheel maps as measured in the macaque^41^ and human^66^.

*Individual receptive fields* were generated using a simple model that additively combines on and off sub-units with spatial separations drawn from a unimodal distribution^45^. The same band-pass filtered white noise that was used to generate orientation and ocular dominance maps was also used to generate the maps governing the separation (δ_on–off_*, Panel D)* and relative strength of receptive on-and off fields (w_on-off_, Panel E) after bandpass filtering at twice the frequency used to generate orientation and ocular dominance columns^46^. We assumed that the contribution of on cells was weighted more heavily than the contribution of off cells (ω_on–off_ = 0.8) enabling us to capture the phenomenon that phosphenes are occasionally dark at threshold, but are consistently bright as current increases above threshold.

Receptive field size was assumed to linearly increase with eccentricity^44^ also see Ref.^67^ (σ_*rf*_, Panel F). Individual example receptive fields for the left and right eye are shown in Panel G, for three exemplar cells.

Electric field spread was modeled based the current-distance equation,$I={I}_{input}/\left(1+K\cdot({rad-{rad}_{e})}^{2}\right),$ where $\text{I}$ is the current in μA at a given location on the cortical surface, ${I}_{input}$ is the stimulating current, and $\text{ra}{d}_{e}$ is the radial distance between that region of the cortical surface and the nearest region of the electrode^68^.

Predicted phosphenes were generated as a linear sum of receptive field profiles at each cortical location, weighted by the current stimulation intensity at that location. We assumed that threshold and brightness are determined by the maximum phosphene brightness over time and space. We assumed that responses reached perceptual detection threshold when the maximum response over time was greater than threshold, $\left(\text{max}\left(resp\right)\ge {\uptheta }_{thresh}\right)$. Phosphene area and shape was quantified, using image moments, after having thresholded the simulated phosphene based on a drawing threshold,$(max(resp)\ge {\uptheta }_{draw})$, to create a binarized image.

### Phosphene thresholds and brightness as a function of the temporal properties of electrical stimulation

**Figure 3:**
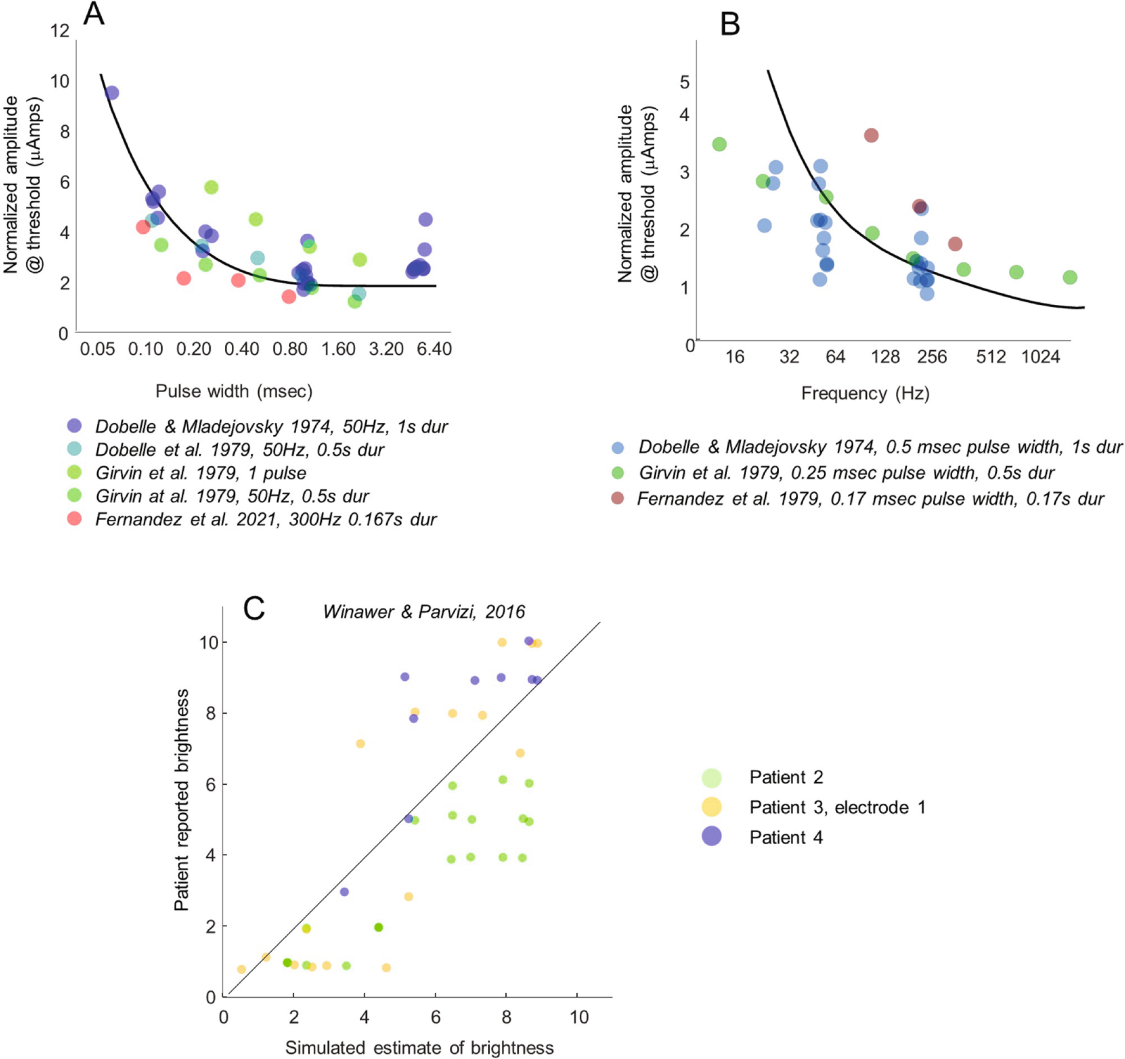
Phosphene thresholds and brightness as a function of pulse train parameters. (**A**) Normalized thresholds as a function of pulse width, (**B**) Normalized thresholds as a function of frequency. Model simulations are shown with black lines. Data points are jittered horizontally (on a log scale) and are transparent for visualization purposes in Panels A and B. (**C**) Predicted brightness as a function of pulse parameters. Each data point represents a single trial. The x-axis represents model predictions based on test data and the y-axis represents patient estimate of brightness on that trial (data points jittered slightly along y-axis). Each color corresponds to a different electrode (the locations of the electrodes on the cortical surface are shown in Fig. 5).

Figure 3 compares model predictions to data measuring current amplitude thresholds (the stimulation current required to reach threshold visibility) and brightness ratings across a variety of pulse trains. Data were normalized across each electrode (note that each of the datasets in Fig. 3A and B contain multiple electrodes, with some electrodes shared across datasets). Normalization was done using linear regression to find the value of *s* that scaled the sensitivity of each electrode to match that of a ‘standard’ electrode. This ‘standard’ electrode was defined as having a 3 μAmp threshold for a 50 Hz cathodic-first pulse train with a pulse width of 0.25 ms and a pulse train duration of 0.5 s (see “STAR methods”).

Figure 3A shows thresholds as a function of pulse width collated from human acute^29^ and chronic surface^32,33^ and depth electrodes^27^. Model threshold predictions for a 50 Hz cathodic-first 0.5 s pulse train with varying pulse durations are shown as a black solid curve. In our model the shape of the function relating threshold as a function of pulse width—the ‘strength-duration’ curve^69^ is entirely determined by the first integration stage of our temporal model, and is independent of electrode size, frequency or pulse train duration. As can be seen in Figure 3A, consistent with our model, given a single scaling parameter, the shape of experimental strength-duration curves showed little variation across a wide range of experimental protocols. There was a strong correlation between model predictions and experimental thresholds: (*r*(43) = 0.804, *p* < 0.0001).

Figure 3B shows thresholds as a function of pulse frequency collated from human acute^29^ and chronic surface^32^ and depth^27^ electrodes. Model threshold predictions for a cathodic-first 0.5 s pulse train with a pulse width of 0.25 and varying frequency are shown as a black solid curve. The shape of the curve relating thresholds as a function of frequency varied across studies, with all three data sets showing different slopes. In our model, threshold as a function of frequency is determined by the second stage of our temporal model and the refractory period. We selected model parameters that captured a slope intermediate between these three studies; nonetheless there was a strong correlation between model predictions and experimental thresholds: (*r*(34) = 0.774, *p* < 0.0001).

Because most reported cortical simulation data consists of relatively short periods of stimulation, we chose not to model desensitization/adaptation as a result of repeated stimulation over several seconds, as has been observed in both the retinal^70^ and cortical literature^34^ (also Dagnelie, personal communication).

Figure 3C compares model predictions to patient apparent brightness ratings (on a 1–10 scale) across pulse trains that vary in pulse width (0.2–1 ms), frequency (5–100 Hz), pulse-train duration (0.2–1 s) and amplitude (0.2–5 mA) in three surface electrodes^35^. There is a strong correlation between simulation predictions and patient data (*r*(42) = 0.771, *p* < 0.0001).

Thus, the data from Fig. 3, collated across a wide variety of studies, supports the notion that a basic model describing the transformation from pulse trains to perceptual intensity over time can successfully predict both thresholds and brightness ratings across a wide range of pulse train parameters, electrode locations and sizes. In practice, the goal of most stimulation protocols is to maximize charge efficiency in order to maximize battery life: our model predicts little benefit for increasing pulse width durations beyond 0.4 ms, or stimulation frequencies above 64 Hz.

### Phosphene size as a function of current amplitude

**Figure 4:**
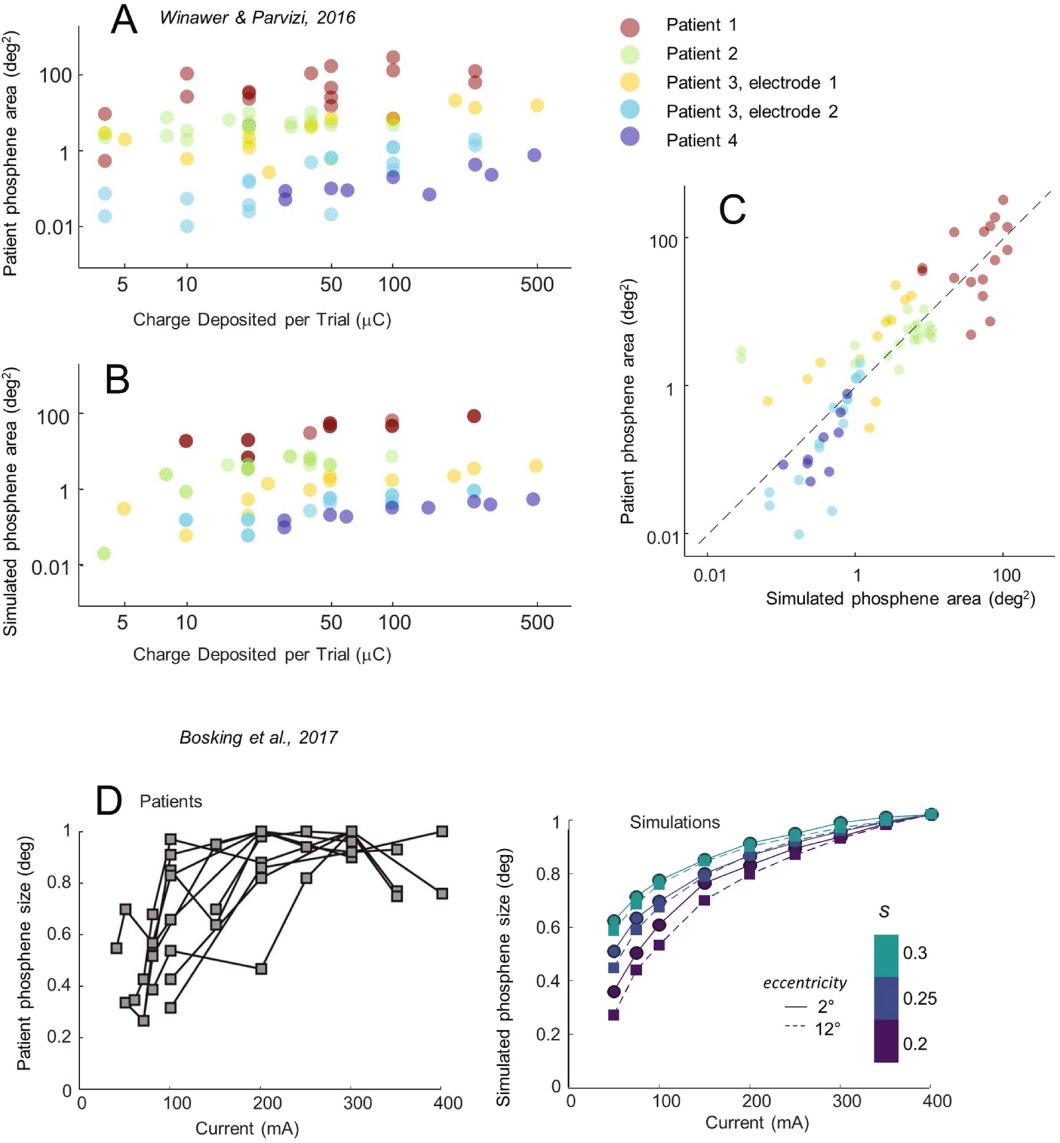
Phosphene size as function of current amplitude. (**A**) Patient reports for phosphene area (based on patients drawing the perceived phosphene with their finger on a laptop touchpad) as a function of total charge per trial. Each color corresponds to a different electrode (the locations of the electrodes on the cortical surface are shown in Fig. 5). Each data point represents a single drawing. Data points are transparent for visualization purposes. Panel replotted from Neuron, 92/6, J. Winawer and J. Parvizi, Linking Electrical Stimulation of Human Primary Visual Cortex, Size of Affected Cortical Area, Neuronal Responses, and Subjective Experience, (**A**), Copyright (2016), with permission from Elsevier. (**B**) Simulation predictions of the data in panel (**A**). (**C**) A direct comparison of simulations vs. patient drawings. (**D**) Left panel shows normalized phosphene size as a function of current amplitude replotted from Bosking et al.^71^ (patient drawings were made using similar methods as Ref.^35^). In this paper, size was reported as the mean of the major and minor diameter of the best-fit ellipse. Right panel shows corresponding simulations for two eccentricities (2 & 12 degrees) and three s values.

Our model also successfully predicts phosphene size as a function of amplitude. Figure 4A–C shows simulations of data from Winawer and Parvizi^35^ examining phosphene size as a function of amplitude, with  Fig. 4A showing patient data and Figure 4B showing model simulations. (We plot data as a function of charge to match the original paper but it is worth noting that identical charge can result in differently sized phosphenes, depending on pulse width and frequency, which is why the data in Fig. 4B has scatter along the y-axis.) C directly compares model predictions to patient report. Once again, there is a strong correlation between simulation predictions and patient data (*r*(77) = 0.833, *p* < p < 0.0001).

**Figure 5:**
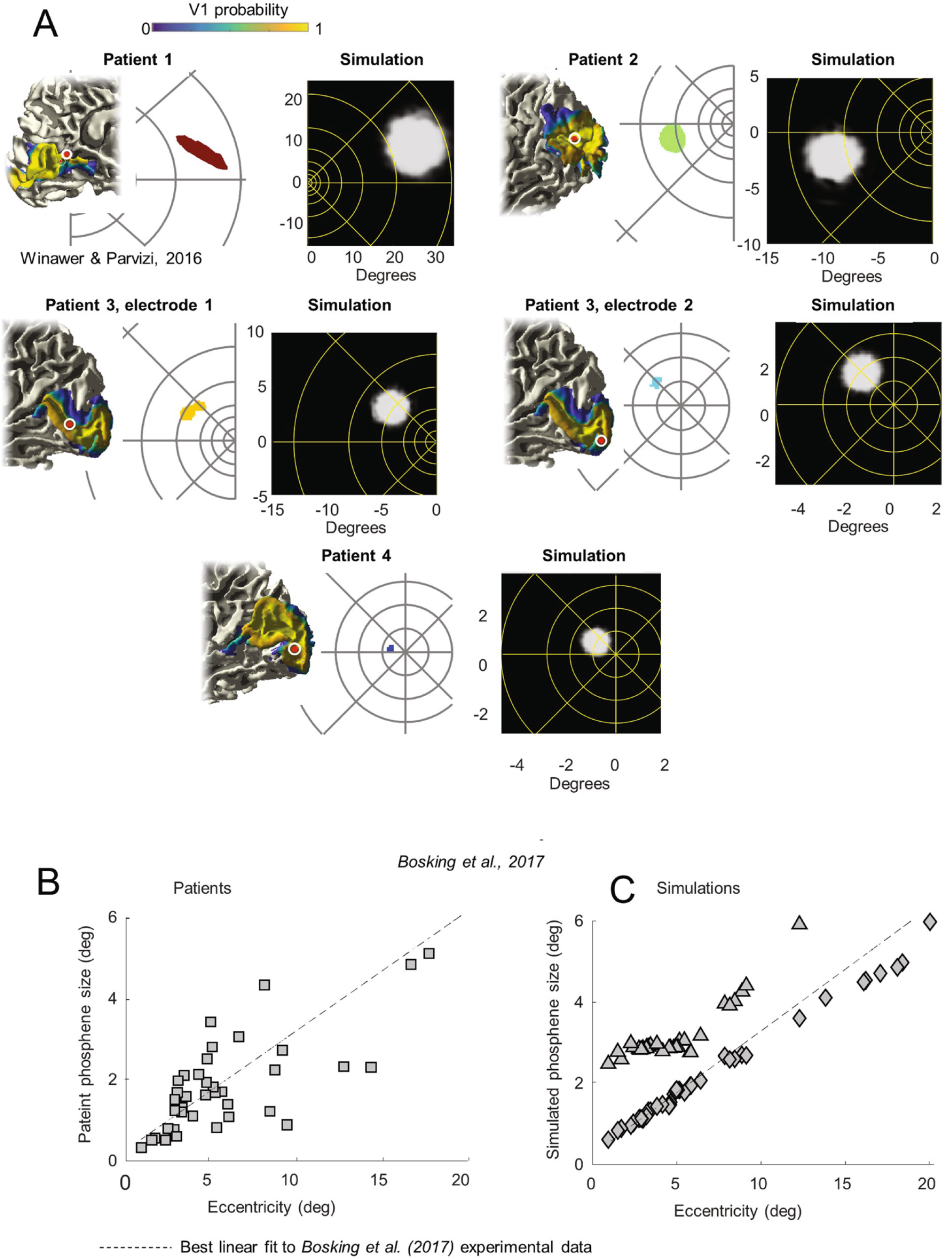
Phosphene size as a function of eccentricity. (**A**) Anatomical images show electrode location (5 locations, electrode locations 3 and 4 are two electrodes in a single patient) overlaid on the probabilistic atlas of V1^72^ applied to each subject’s T1-weighted anatomical MRI. Estimated electrode position is shown as red circles, with the white circle indicating positional uncertainty of 5 mm in radius. Panel replotted from Neuron, 92/6, J. Winawer and J. Parvizi, linking electrical stimulation of human primary visual cortex, size of affected cortical area, Neuronal Responses, and Subjective Experience, Fig. 1A, Copyright (2016), with permission from Elsevier. All electrodes are within high probability areas of the Hinds V1. The white panels show single typical phosphene drawings for the 5 electrodes (replotted from Ref.^35^), while the black panels show the corresponding simulated phosphenes. Eccentricity lines are drawn at 1, 2, 3, 5, 8, 13, 21, and 34° in both panels. (**B**) Phosphene size as a function of eccentricity replotted from Ref.^24^. (**C**) simulated data for the same eccentricities. Diamond symbols represent predictions based on a single study macaque electrophysiological receptive field size estimates^44^ and triangle symbols represent predictions based on a meta-analysis of five studies of V1 macaque receptive field sizes^67^. The dashed line in panels B and C represent the best linear fit to the Bosking et al. patient data.

Figure 4D shows model and simulated predictions for data by Bosking et al.^24^ examining phosphene size (normalized to the maximum size) as a function of current amplitude. Very similar amplitude-size functions have also been observed by Fernandez et al.^27^. Once phosphene size is normalized to the maximum size, the effect of eccentricity (difference between square and circle symbols) on the curves relating phosphene size to stimulation amplitude is small. Our simulations suggest that electrode sensitivity (*s*) may play a more major role in influencing the current amplitude-size curve.

### Phosphene size as a function of eccentricity

Our model successfully predicts the finding that phosphene size increases as a function of eccentricity in the visual field. Figure 5A shows simulations based on patient drawings made for five surface electrodes in four patients^35^. Electrode radii were 0.575 mm for the electrode of patient 2, and 1.15 mm for the remaining four electrodes. Our model captures the phenomenon whereby phosphene size increases with eccentricity (note the large change of scale across panels).

Our model also replicates data from Bosking et al.^71^ who examined phosphene size as a function of eccentricity for 93 surface electrodes (0.25 mm radius) implanted in 13 patients. Figure 5B replots estimates of phosphene size based on patient drawings, with the dashed line showing the best linear fit, (*r*(41) = 0.884, *p* < 0.0001). Figure 5C shows simulated predictions for these same eccentricities, along with replotting the best linear fit to the Bosking et al. patient data. The upper curve (triangles) in Figure 5C represents estimates based on a meta-analysis^67^ of five older published studies in cebus, owl and macaque monkeys^73–77^. The lower curve (diamonds) is a simulation based on estimates of receptive field sizes made in a single macaque by Keliris et al.^44^: these were the smallest estimates of receptive field sizes that we found in the published literature. Simulations based on the Keliris et al. data were well correlated with the patient data, (*r*(41) = 0.880, *p* < 0.0001). The very small differences between patient data and our simulated predictions based on might easily be due to species differences^78,79^, individual differences^80^, or measurement sampling.

Unsurprisingly, given that receptive field sizes are thought to be inversely related to cortical magnification (millimeters of cortex per degree of visual angle)^81,82^, our model predictions are similar to those made by others^24,83^ using simpler models that assume that phosphene size is inversely proportional to cortical magnification.

### Shape recognition

Previous experimental studies have found it extremely difficult to generate recognizable shapes through stimulation of multiple electrodes^34,84^. Recently, Beauchamp et al.^25^ showed that subjects can identify simple forms when multiple electrodes are stimulated in sequence even though those same shapes are uninterpretable when electrodes are simultaneously stimulated.

**Figure 6:**
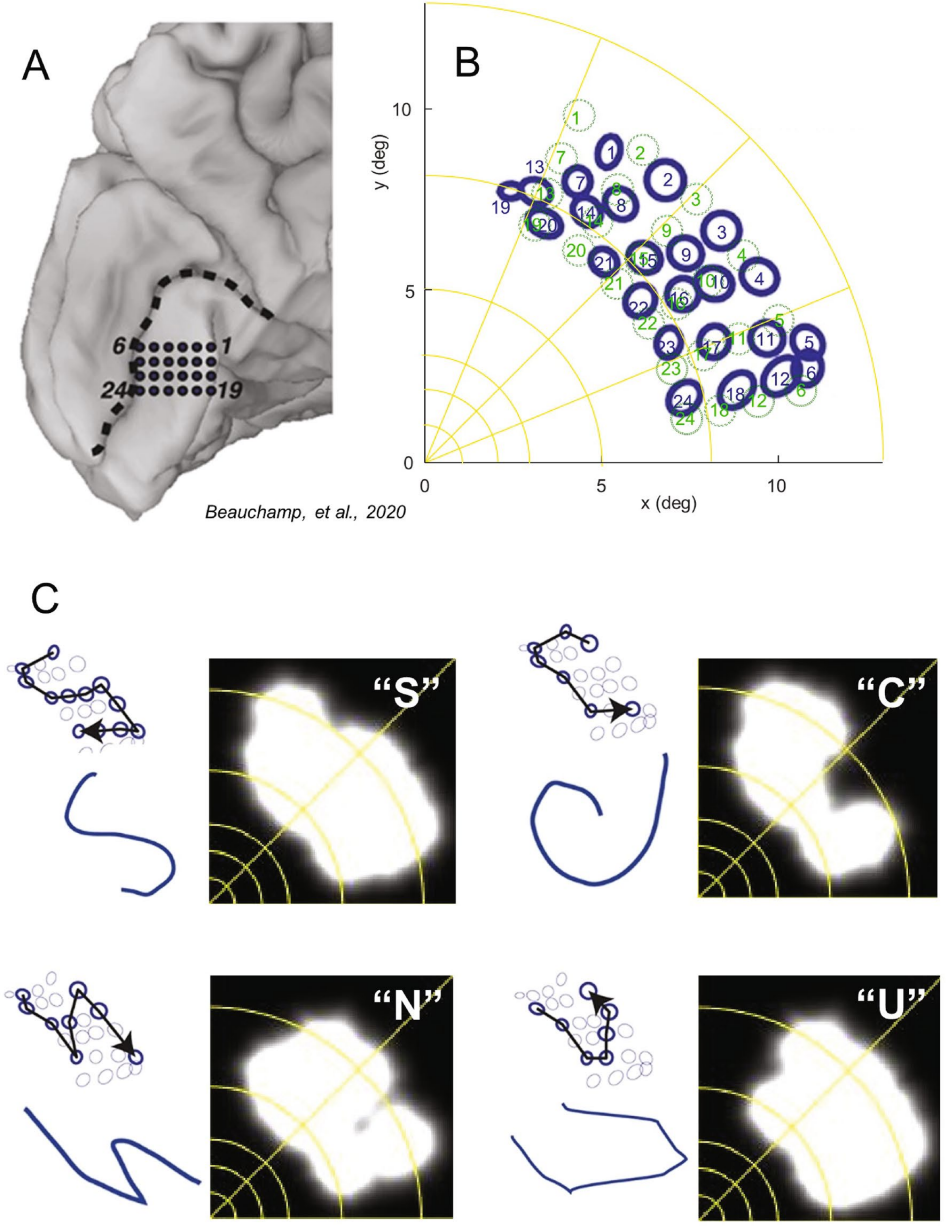
Shape recognition for multiple electrodes. (**A**) Medial view of the left occipital lobe of a sighted patient. Black dots show the 24 electrodes contained in a grid implanted inferior to the calcarine sulcus (dashed black line), replotted from Beauchamp et al.^25^. (**B**) The patient fixated while electrodes were stimulated and then drew the perceived location of the phosphene with their finger. The blue circles replot ‘phosphene maps’—the drawn location in visual space for each electrode^25^. Green circles show simulated predicted phosphene locations based on estimating the location of the cortical grid on the cortical surface. (**C**) Beauchamp et al.^25^ stimulated selected electrodes to generate four different “letter” percepts. Electrodes in each trajectory were stimulated with small amounts of current (~ 1 mA) at high frequency (~ 200 Hz) either simultaneously or in rapid temporal sequence (50 ms per electrode, 50 ms delay between each electrode). For each ‘letter’, the upper left panel replots the patient reported phosphene maps of stimulated electrodes (bold circles) and the direction of the temporal sequence of stimulation (arrow). The lower panel replots the participant’s actual drawing of the visual percept. The right panels show our model predictions for simultaneous stimulation (for sequential simulation, see Supplementary Videos (LettersEstimatedLocation.mov—Supplementary Video 5, LettersPatientLocation.mov—Supplementary Video 6) for Fig. 6). Panel (**A**–**C**) modified from Cell, 181/4, M.S. Beauchamp, D. Oswalt, P. Sun, B.L. Foster, J.F. Magnotti, S. Niketeghad, N. Pouratian, W.H. Bosking, D.Yoshor, dynamic stimulation of visual cortex produces form vision in sighted and blind humans, Fig. 4, Copyright (2020), with permission from Elsevier.

If one compares the prediction from simultaneous stimulation (Fig. 6) to simulations based on sequential stimulation (See Supplementary Videos (LettersEstimatedLocation.mov—Supplementary Video 5), a critical aspect of the patient data is revealed – letter shapes are not interpretable using simultaneous stimulation, but are interpretable using sequential stimulation. Because our model does not include electrical or complex neuronal spatiotemporal interactions these results suggest that the primary difficulty with simultaneous stimulation may be due to a ‘Gestalt’ failure to correctly group phosphenes. As shown in Fig. 6, our model, based on a prediction of phosphene locations based on aligning the electrode array to a cortical anatomical model (see “STAR methods”), produces perceptual predictions that are very close to patient reports (see LettersPatientLocation.mov, Supplementary Video 6 for predictions based on patient reports of electrode locations, which are qualitatively very similar).

### Using ‘virtual patients’ to predict perceptual outcomes for novel devices

Our ability to replicate such a wide range of data suggests that this model is capable of providing insight into the likely perceptual experience of *novel* technologies—one of the more important uses of ‘virtual patients’.

**Figure 7:**
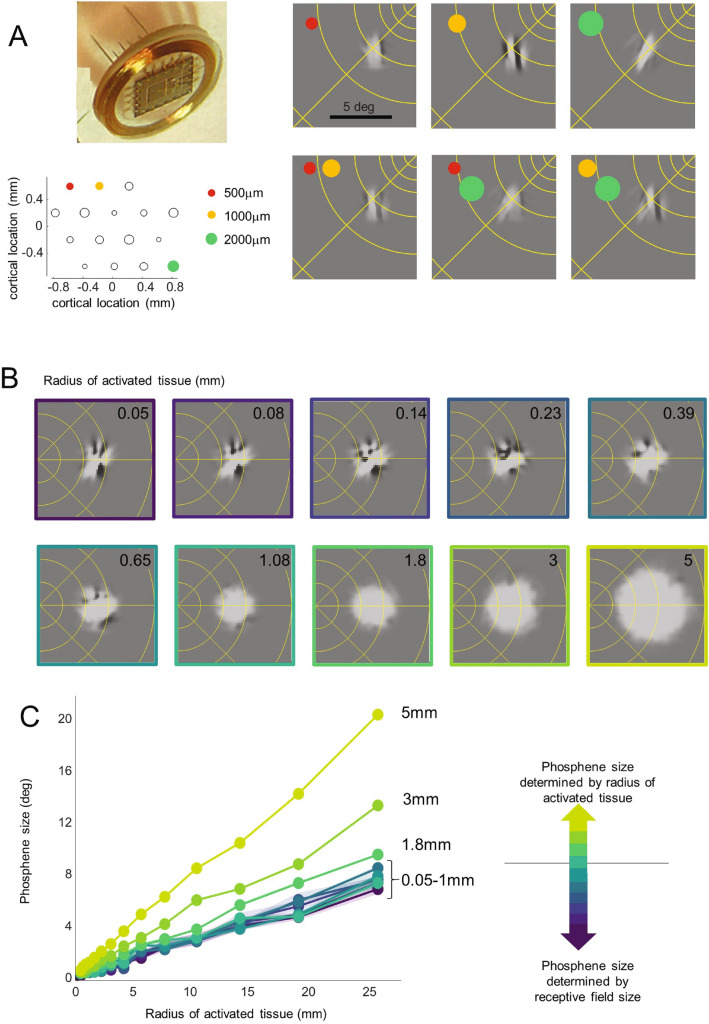
Using virtual patients to predict perceptual outcomes. (**A**) Simulated percepts for an array containing very small depth electrodes^85^, array image and informal observations kindly supplied by P. Troyk and G. Dagnelie. The locations and sizes of electrodes in the array are shown in the lower left panel. The upper right panels show example percepts for the three individual electrodes (assuming the array was centered on a cortical region that represents 5° eccentricity). The lower panels show predictions when simultaneously stimulating paired combinations of electrodes. (**B**,**C**) Simulated predicted percept shapes and sizes across a range of electrode sizes and cortical locations. The narrow shaded regions in Panel C represent 5–95% confidence intervals. Simulations were based on receptive field size estimates from Keliris et al.^44^.

Figure 7A shows predicted phosphenes for extremely small electrodes near the fovea, using a simulated array based on a prosthetic device with extremely small (tip areas between 500–2000μm^2^) depth electrodes, replicating a device that is in the very early stages of a clinical trial^85^. The only alteration we made to our model was to assume that depth electrodes result in extremely narrow current spread (K = 10^5^ μA/mm^2^). The upper panel shows simulations for individual electrodes, and the lower panel shows simulations for paired stimulation. Consistent with preliminary data^86^, our simulations predict that nearby electrodes are not spatially resolvable. Our simulations are consistent with informal experimental observations in patients that stimulation of individual or multiple electrodes separated by 0.4–1.85 mm in cortex result in irregularly shaped (“amoeba” or “crosses”) phosphenes of roughly a half degree in size that contain dark regions. Our predictions reflect the fact that orientation and on–off dominance columns are relatively large (> 2 mm for a full ocular dominance/pinwheel map^41,42^). As a result, stimulation with extremely small electrodes will potentially stimulate neurons tuned for similar orientations, creating percepts that are elongated, or have complicated structure.

Figure 7B and C examines the predicted effect of electrode size on patient percepts. For small electrodes with limited current spread, resulting in less than ~ 0.25 mm radius of cortical tissue being stimulated, phosphenes tend to have a complicated structure (upper panels of Panel B) and the size of the electrode has little effect on the appearance or size of the percept. Between 0.25 and 1 mm, the phosphenes begin to approximate a “Gaussian blob”, but the size of the phosphene is still primarily determined by receptive field sizes rather than the extent of stimulated tissue. It is not until electrodes have radii above 1 mm that the size of the electrode has an appreciable impact on the size of the phosphene. Critically, our simulations suggest that, across the entire visual field, receptive fields impose a neurophysiological ‘lower limit’ on phosphene size. Reducing the radius of stimulated tissue below 0.5 mm may have little benefit for acuity and may result in less interpretable phosphenes.

**Figure 8:**
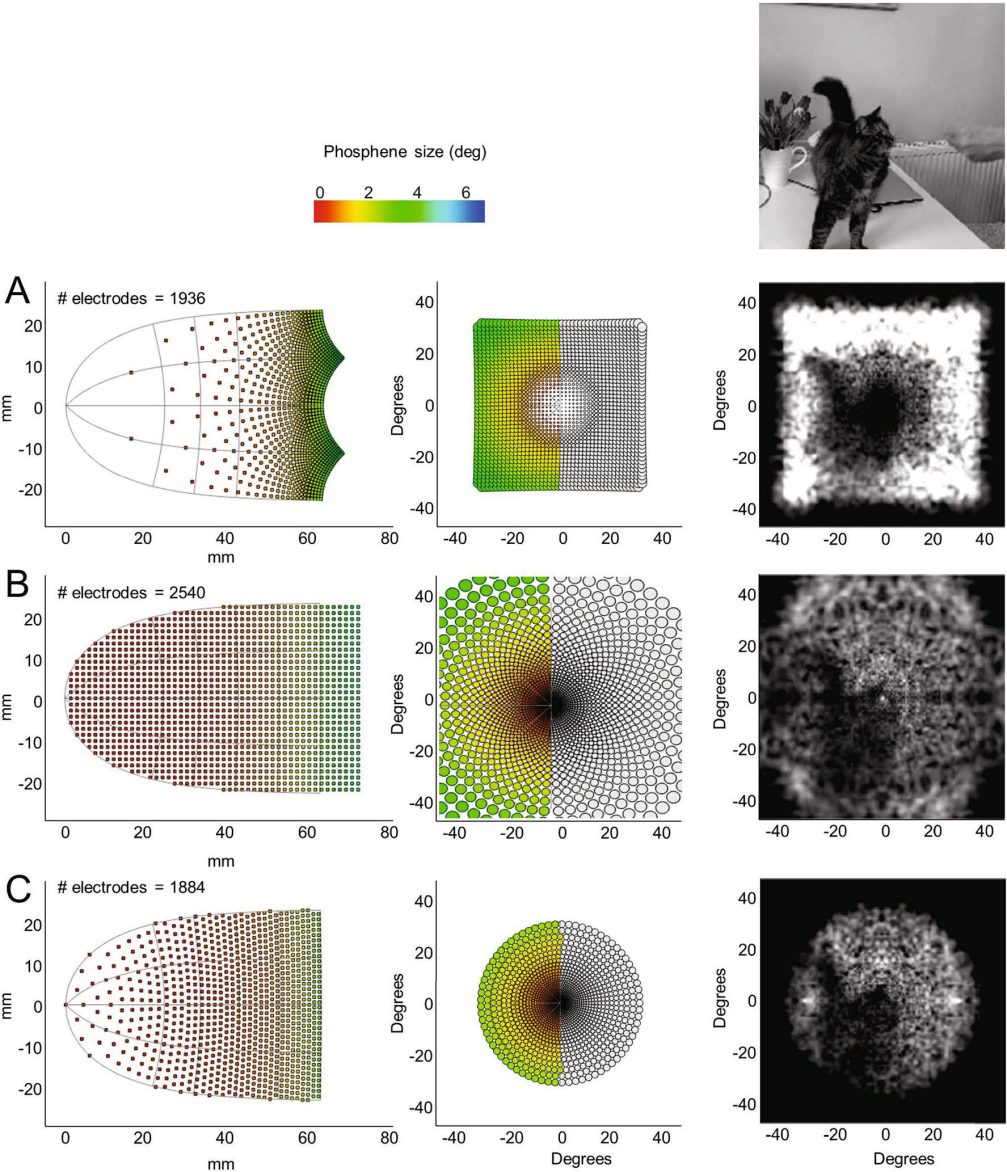
Simulations comparing different electrode array configurations. The left panel shows the electrode placement on the cortical surface of V1, the middle panel shows simulated phosphene sizes as a function of eccentricity in visual space co-ordinates. The rightmost panel shows a single image from a simulated movie (see Supplementary Fig. 8 (ArraySimulations_Spacing_1.mov—Supplementary Video 1, ArraySimulations_Spacing_2.mov—Supplementary Video 2, ArraySimulations_Spacing_3.mov—Supplementary Video 3, ArraySimulations_Spacing_4.mov—Supplementary Video 4) for the full movies). (**A**) Regular spacing of electrodes on the visual field. (**B**) Regular spacing of electrodes on the cortical surface. (**C**) ‘Optimal’ spacing (the center-to-center separation of phosphenes is a constant proportion of phosphene size). See Supplementary Videos (ArraySimulations_Spacing_1.mov—Supplementary Video 1, ArraySimulations_Spacing_2.mov—Supplementary Video 2, ArraySimulations_Spacing_3.mov—Supplementary Video 3, ArraySimulations_Spacing_4.mov—Supplementary Video 4) for Figs. 8, which also includes simulations for lower density arrays.

Figure 8 shows simulated perceptual outcomes (also see Supplemental Video (ArraySimulations_Spacing_1.mov—Supplementary Video 1, ArraySimulations_Spacing_2.mov—Supplementary Video 2, ArraySimulations_Spacing_3.mov—Supplementary Video 3, ArraySimulations_Spacing_4.mov—Supplementary Video 4) for Fig. 8) for three electrode array configurations. The number of electrodes were chosen to be roughly similar across arrays, while compensating for slight differences in the area of visual field represented. Figure 8A shows electrodes arranged to produce a regular tiling in visual space. This array clearly underrepresents the fovea – producing a sparse collection of tiny phosphenes in the fovea.

Figure 8B shows electrodes arranged regularly on the cortical surface. This configuration suffers from the opposite problem—an over-tiling of electrodes within the fovea, producing a cluster of heavily overlapping receptive fields within the fovea, as has been informally observed in one recent study^86^. These overlapping receptive fields seem to offer little to no benefit in terms of resolution: In the region of cortex representing the foveal confluence nearby electrodes project to almost the same location of visual space – so small shifts in electrode locations on the cortical surface produces imperceptible shifts in phosphene location relative to the size of foveal phosphenes. Thus, the assumption often made in the cortical stimulation literature, that the massive expansion of the foveal representation in V1 might allow for relatively high sampling of spatial position, is probably incorrect.

Finally, Fig. 8C shows an ‘optimal’ configuration where electrodes are spaced such that the center-to-center separation of the elicited phosphenes is a fixed factor of phosphene size. Because receptive fields vary linearly with eccentricity^67^ while cortical magnification varies logarithmically^64,82^, the optimal configuration packs electrodes less tightly in regions of the cortex representing the fovea than regions representing the periphery (see “STAR methods”). Although this configuration actually had the smallest number of electrodes, the image is perceptually the most interpretable (see below for a discussion of how image perceptual quality might be more rigorously quantified). Thus, our simulations suggest that electrodes should actually be placed more sparsely in regions of cortex representing the foveal confluence – the opposite of the common intuition that electrodes should be placed more densely in foveal regions. The lack of benefit of increasing the number of electrodes in the fovea is counterintuitive. However, as discussed more fully below, our ability to see in high resolution is based on interpreting the complex pattern of responses across a population of neurons with receptive fields tuned to multiple locations, orientations and sizes.

More generally, simulations such as these allow one to predict the best possible perceptual performance for any given array, based on neurophysiological constraints.

## Discussion

Despite its simplicity and lack of fitted parameters, our model successfully predicts a wide range of psychophysical and electrophysiological cortical stimulation data. One of the reasons our model generalizes so successfully is because it is spatiotemporally separable: the perceptual effect of the temporal properties of the pulse train (e.g. pulse width and frequency) is independent of the spatial properties of electrode location and, electrode size.

Whenever possible (see Table 1) we based parameters on independent datasets describing V1 architecture. The only factor that was allowed to vary across experiments was a sensitivity parameter (*s*) that linearly scaled current amplitude. This parameter likely mediates the effects of a multitude of factors affecting sensitivity including the distance of the electrode from the cortical surface, electrode size (which alters current density), and the distribution of current over the electrode surface.

### Neurophysiological basis of the model

The relationship between extracellular stimulation, neuronal depolarization and spiking thresholds has been modeled at various levels of complexity, including finite element simulations^57^ and simulations that treat cells as a network of resistances and capacitances (compartment models)^58,59^. We used a simpler well-established model which assumes that the activating function (the rate of membrane potential change if the neuron is in resting state before the stimulation) is proportional to the second-order spatial derivative of the extracellular potential over the cell^55,56^. This was modeled by a one-stage leaky integrator, for which the rate of change of depolarization is proportional to the current level of depolarization plus the input current. It is well established that this one-stage leaky integrator provides a reasonable approximation to cellular depolarization due to extracellular stimulation^60–62^.

At a single cell level, once depolarized to ‘threshold’ a neuron will (almost always) produce a single spike rather than multiple spikes (since the pulse durations used in electrical stimulation are short compared to the refractory period). Thus, at a single cell level, once a spike is elicited, there is little effect of increasing the current amplitude further.

However, because the capacitance and resistance of individual neurons vary, based on their size, shape and myelination, neurons vary in the sensitivity of their activating function. Consequently, as the current amplitude of a pulse increases, gradually more and more cells under the electrode will reach their depolarization threshold. Thus, in our model, the output of the first stage, ‘spike response strength’ should not be thought of as representing spikes per se, but as reflecting the recruitment of spikes from a population of cells with activation functions that vary in sensitivity. With increasing amplitude, the neural response directly under the electrode will eventually saturate, so the maximum brightness will eventually asymptote. Our model includes a response compression stage, which likely captures both saturation within this population of cells, and the effects of more complex cortical gain control mechanisms. (Because our model is linear with respect to response strength up to the non-linearity, the response compression was placed as the final stage of the model for computational convenience. However, it is plausible that this response compression may occur earlier in the pathway).

However, increases in current amplitude also cause cells further from the electrode to reach their depolarization threshold, due to current spread, increasing the size of the phosphene.

Supplementary Fig. 1 shows simulations showing the differential effect of increasing frequency vs. amplitude on apparent brightness and phosphene size as a function of pulse train frequency and amplitude.

### Limitations of the model

Obviously cortical stimulation involves a vast range of engineering and neurophysiological complexities that are not captured by our model. A subset of these are described below.

First, our model uses current amplitude as input. Theoretically, we should have used current density rather than current amplitude. However in practice current “pools” around the edge of electrodes^87,88^ – for the large electrodes used in some of the studies we simulated, current intensity can be five-fold higher at the edge of the electrode than in the middle.

Second, we assume that electrodes are flush to the cortical surface. In practice, electrodes are unlikely to be flush to the surface, and even a slight tilt of the electrode relative to the cortical surface is likely to result in only the edge of the electrode being effective in driving a neuronal response.

Third, although our model fits current temporal data reasonably well and includes desensitization due to the refractory period, it is worth noting that various studies have shown longer-scale desensitization with prolonged stimulation in both human^34^ and macaque^89^ and this is also observed with retinal prostheses^70^. Our model should therefore be considered an approximation that will not generalize to longer stimulation protocols.

Fourth, our model does not include either electric field or nonlinear neural interactions. Despite this, our model does replicate previous findings that simultaneous stimulation results in less interpretable percepts then sequential stimulation. Nonetheless, it remains probable that electrical and neuronal interactions play an additional complicating role^54^. Previous work, with retinal implants, has demonstrated that both electric field interactions and rapid neural integration (on the order of 3–9 ms) influence patient percepts^90^.

Fifth, our model assumes that percepts are generated as a simple average of each receptive field. An alternative approach is to assume that each neuron is better characterized in terms of its ‘optimal reconstruction filter’—the contribution of that cell to the reconstruction of a natural image in the context of a neural population^91^. However, at the retinal level it has been found that as experimental sampling of the population of neurons converges to a complete tiling, the reconstruction filters generated by this approach come to resemble receptive fields as measured using white noise stimuli. Thus our linear averaging may, in practice, capture the essential structure of how neuronal messages are encoded by the brain^91^.

Finally, our current model only includes cortical area V1. Because of the configuration of the cortical surface, it is much easier to implant electrodes in higher-level visual areas such as areas V2 or V3. Many aspects of our model, including the transformation from visual space to cortical surface^63,65,92^, will easily generalize to these higher visual areas. Our model could also be easily generalized to incorporate models of V2 or V3 neuronal receptive fields. However, the complexities of V2-V3 neural receptive field structure, along with the lack of cortical stimulation data from electrodes identified as being in V2 or V3, means that for the time being any such generalization of the model would be extremely speculative.

### Insights from the model

Our model predicts that three main factors limit the spatial resolution that can be provided by cortical electrical implants: cortical magnification, receptive field structure, and the size of the electrode.

Receptive field sizes have a close relationship with cortical magnification. Across much of cortex receptive field areas approximate the areal cortical magnification to the − 2/3 power^82^; explaining the previous observation of Bosking et al.^24^ that the size of the phosphenes drawn by their patients could be predicted by cortical magnification. At the fovea, cortical magnification reaches a maximum, and therefore receptive field sizes reach a minimum, somewhere between 0.02 and 0.5 degrees in radius.

Both data and simulations suggest that, for a fixed electrode size, phosphene size increases linearly as a function of eccentricity. For electrode radii less than 0.25 mm this linear relationship is primarily due to the increase in receptive field sizes as a function of eccentricity. It is only for larger electrode sizes that cortical magnification and the extent of stimulated cortex also plays a role.

For smaller electrodes, it is receptive field sizes that limit acuity. Our optimal spacing calculations are based on estimated average receptive field sizes. If it were possible to selectively stimulate neurons with very small receptive fields, the optimal electrode spacing would be packed more tightly in the fovea, and higher resolution could be obtained.

However, it is important to note that humans can resolve spatial detail (Vernier acuity of 0.3–1 min arc^93^, and grating acuity of ~ 50–60 cycles/degree, much smaller than single receptive fields: Indeed, humans have the ability to detect spatial offsets smaller than the width of a single cone^94^. Our ability to perceive a single point of light, a fine grating, or the offset in a thin line is not limited by minimum receptive field sizes. Extremely fine spatial discriminations are based on interpreting a complex pattern of responses across a population of neurons with different receptive fields. An insight from Fourier analysis, that may or may not help, is that an impulse contains a *flat* spatial frequency spectrum. Thus, a discrete point of light  - a single star - contains an infinitely broad range of spatial frequencies. If one interprets the responses of early visual areas as being approximate to a wavelet analysis, one would expect the resolution of small spots of light to be mediated by a population response across hundreds or thousands of V1 neurons with a wide range of receptive field shapes and sizes. Conversely, generating a punctate percept through electrical stimulation would require appropriately stimulating many hundreds or thousands of neurons^95^.

Overall, our simulations suggest that neurophysiological rather than engineering constraints are likely to limit the spatial resolution of cortical prostheses for the foreseeable future.

### Virtual patients

Models like ours can be considered ‘virtual patients’ and play a role similar to that of virtual prototypes or digital manufacturing. This work is conceptually similar to a previous virtual human patient for electronic retinal prostheses^48,96^, that has been used by both research groups and prosthetic companies as a research and design tool.

Virtual prototyping (also known as digital manufacturing) has revolutionized the design of complex engineered systems such as airplanes, and analogous techniques of biological simulation are rapidly becoming critical for drug development. Comparable modeling techniques have long used to model the effect of electrical stimulation on local tissue, e.g. the current spread for an electrode^97–99^. However, without extending virtual prototyping to include the basic physiology of early visual areas it is impossible to predict perceptual outcomes. Our simple model is successful at predicting a wide range of cortical electrical stimulation results, suggesting that it is likely to provide a reasonable approximation of predicted perceptual outcomes for future implants.

Virtual patients like ours are critical for solving a fundamental issue for sight restoration development – *it is currently impossible to predict outcomes before implanting in humans*. Currently, the neural implant field relies on intuition and iterative trial-and-error – a process unnervingly similar to the earliest days of aviation*.* A cursory web search for images related to “aviation 1890” makes it clear that many of the perfectly logical intuitions of engineers of the period were deeply mistaken. Our model suggests that analogous intuitive fallacies are currently influencing the field of cortical electrical stimulation. One such fallacy is the intuition that smaller electrodes will always result in smaller percepts: our model suggests little increased benefit for electrodes sizes below 0.25mm radius across much of the visual field. Another is the notion that the massive expansion of the foveal representation in V1 should be exploited to improve resolution. Our simulations suggest the opposite: that electrodes should be placed either uniformly or more sparsely in the foveal than peripheral cortex.

For researchers and companies, these models can provide a variety of uses. One is to provide a quantitative test of whether we have a full understanding of the technology. Given the difficulty of collecting behavioral cortical data^71^, a model driven approach is likely to be useful in determining which experiments will gain the most useful insights.

A second important use of the virtual patient is to predict the quality of vision likely to be produced by a given implant. In this paper we relied on qualitative evaluation of perceptual quality when assessing different array configurations. One more rigorous approach is to focus on subjective interpretability: through having normally sighted individuals perform perceptual tasks using simulated prosthetic vision^100–102^. Another approach is to use simulations as input images for a decoder that is trained to generate a reconstruction of the original input image, as has been done recently using a cortical simulator that approximates some of the same phenomena as our more elaborated model^83^.

Finally, these virtual patients can guide new technology development. For example, as described above, our current model counterintuitively suggests limited advantages to small electrode sizes and dense implantation in regions of cortex devoted to the fovea. Virtual patients can also be used to generate training sets for deep learning-based prosthetic vision optimization designed to find the best stimulation pattern for existing implants. An analogous model of retinal stimulation is currently being used to simulate and optimize prosthetic vision in an VR setting^103^ by generating training sets for deep learning-based preprocessing^104^.

For entities such as the FDA and Medicare, these models can provide insights into what sorts of visual tests/metrics will be important for evaluating devices. Finally, for surgeons and patient families, these models will provide more realistic expectations of likely perceptual outcomes.

## STAR methods

### Lead contact and materials availability

All data used in this paper were publicly available. Data are taken from a variety of papers. Summary data values used for modeling are included in the GitHub repository containing the model (https://github.com/VisCog/p2p-cortical).

### Experimental model and subject details

Data are from human. Experimental values relevant to modeling (e.g. electrode size and location) are included in the GitHub repository. Subject details not included in the model (e.g. sex, age) are described in the associated primary papers.

### Method details

#### Electric field spread

The spread of current in cortical tissue was modeled as follows:

$$spread = \left\{ {\begin{array}{*{20}c} {rad \le rad_{{e}}, I = I_{{input}} } & {} \\ {rad > rad_{{e}} ,I = \frac{I_{{input}}}{ {1 + K\cdot(rad - rad_{e} )^{2} } }} \\ \end{array} } \right.$$

where ${I}_{input}$ is the stimulating current, $\text{I}$ is the current at a given location on the cortical surface, $\text{rad}$ is the radial distance between that region of the cortical surface and the center of the electrode, and $\text{ra}{d}_{e}$ is the electrode radius. For surface electrodes we used a value of K = 675 μA/mm^2^, based on previous estimates of Tehovnik et al.^68^.

#### Transformation from pulse trains to perceptual intensity over time

Our temporal model was loosely based on a previous model originally designed to model epiretinal stimulation^48,70,105^. The first stage of the model is a one-stage leaky integrator so that for a stimulus time-course of current *p(t)* and time constant, *τ*_*1*,_ the response of the first linear stage, *R*_*1*_*(t)*, can be described by the first order linear differential equation:

$$\frac{d{R}_{1}}{dt}=\text{p}\left(\text{t}\right)-\frac{{\text{R}}_{1}}{{\uptau }_{1}}.$$

For example, the response to the onset at time zero of a constant current of amplitude *A* will be:

$${\text{R}}_{1}\left(\text{t}\right)=\text{A}{\uptau }_{1}\left(1-{e}^{-\frac{t}{{\uptau }_{1}}}\right).$$

Our simulations fixed ${\uptau }_{1}=0.3 \text{m}$ s, based on Nowak and Bullier^54^. This rapid, linear, integration stage can be considered to reflect cellular integration of current.

The second stage is the estimation of ‘spike response strength’ whenever *R*_*1*_ peaks. For the standard biphasic pulse trains used in our simulations, we assume that neural spiking occurs at the offset of the positive phase of each biphasic pulse.

We assume that the spiking response strength is attenuated as the inter-spike interval decreases, consistent with known refractory periods in V1. Let *t*_*i*_ be the time at spiking activity event *i* and Δ_i_ be the inter-spike interval, Δ_i_ = *t*_*i*_*–t*_*i-1*_, then the spiking response strength *S* at time *t*_*i*_ is:

$$\text{S}\left({\text{t}}_{\text{i}}\right)={\text{R}}_{1}\left({t}_{i}\right)\left[1-{e}^{-{\tau }_{r}\left({\Delta }_{i}+\delta \right)}\right]$$

where *τ*_*r*_ is a time constant and δ is a constant that sets the minimum amount of inter-spike interval attenuation. S is set to zero during the inter-spiking event intervals. The attenuation due to the inter-spike interval has little effect for low frequency stimulation but reduces spiking strength for frequencies above 50 Hz. We set *τ*_*r*_ = 50 ms and *δ* = 1 ms so that, for example, the attenuation of the average spiking rate drops by 65% for 50 Hz stimulation: $1-{\text{e}}^{-50\cdot \left(\frac{1}{50}+.001\right)}=0.6501$

The third stage is a slow temporal integration stage that converts the rapid spike-events time-course *S(t)* to a slowly changing ‘memory’ of previous spike history. This is computed as a linear convolution of S*(t)* with an impulse response function *G(t)*:

$${\text{R}}_{2}\left(\text{t}\right) = \text{S}\left(\text{t}\right)*\text{G}\left(\text{t}\right)$$

where * denotes convolution. *G(t)* is the impulse response function of an n-stage leaky integrator. *G(t)* is a gamma function defined as:

$$\text{G}(\text{t})={\left(\frac{\text{t}}{{\uptau }_{2}}\right)}^{\text{n}-1}\cdot \frac{{\text{e}}^{-\frac{\text{t}}{{\uptau }_{2}}}}{{\uptau }_{2}\cdot \left(\text{n}-1\right)!}$$

where τ_2_ is a time constant, and n is the number of cascades. We set $n=3,{\uptau }_{2}=150 \text{ms}$. Most parameters were based on the final stage of a previous model describing the effects of electrical retinal stimulation^47^ and τ_2_ was fit based on data examining brightness as a function of frequency, pulse width and pulse train duration^35^.

The final stage is a static compressive nonlinearity defined as a scaled hyperbolic tangent function:

$$brightness\left(t\right)=p\cdot tanh\left(\frac{s{R}_{2}\left(t\right)}{p}\right)$$

The parameter p determines the asymptotic maximum and the parameter s determines the maximum slope of the static nonlinearity (when R_2_ = 0). Based on the fact that the brightness data we used for our model^24,35^ was based on a rating scale (0 when the percept was invisible, 1 for the dimmest visible percept, and 10 for the brightest possible reportable value), we set* p* = 10. Thus, the relationship between neural response and brightness is linear for small values of R_2_ and never exceeds a value of 10.

The parameter* s* was allowed to vary between experiments. Note that although* s* was positioned at the last stage of the model, the model is linear up to this point, so *s* also captures attenuation of current at early stages of the model.

#### Normalizing sensitivity

Electrodes differ considerably in their sensitivity, based on size and how close they are to the cortical surface. We used a single free scale parameter to scale sensitivity for each electrode across all simulations. A default scaling value of *s* = 1 was used for the qualitative simulations of this paper unless otherwise stated.

For simulations where we compared our model to patient data, the scaling parameter, *s*, was estimated in two ways.

In Fig. 3A and B (thresholds as a function of pulse width and frequency) we used a regression procedure, where we defined our neural threshold as the simulated response to a ‘standard’ electrode, defined as having a 3 μAmp threshold for a 50 Hz cathodic-first pulse train with a pulse width of 0.25 ms and a pulse train duration of 0.5 s, using our default scaling value of 0.43. We then calculated expected current amplitude thresholds for this standard pulse train as either pulse or frequency varied, to generate the black line in Fig. 3A and B. For each electrode, we then used linear regression to find the scale factor, *s,* that minimized the difference between these predicted and actual experimental thresholds (when the same electrodes were used to measure threshold as a function of pulse width and frequency a single scaling factor was used across both experiments). Note that this method of linear regression was only possible for threshold data, because our neural threshold was within a response range where the influence of the power nonlinearity was negligible.

Figures 3C (brightness as a function of pulse parameters) and 4B-C (area as a function of pulse parameters) estimated scaling (*s* = *0.57*) and power (*p* = *15.6*) values were estimated using function minimization^35^. (The data from Fig. 3A and B were then refit using these slope and power values: this had no discernable influence on estimates of τ_1_ or τ_2_.) Finally, Fig. 4D, area as a function of charge^24^, was fit using a range of scaling factors.

#### Visual space to cortical surface

The transformation from visual space to the cortical surface was defined using a template derived from a conformal map developed by Schwartz^40,63,64^. Two-dimensional visual space is projected onto the two-dimensional flattened cortex as follows: $\text{w}=\text{k}\cdot l\text{og}\left(z+a\right)$, where $\text{z}$ is a complex number representing a point in visual space, the complex value $w$ represents the corresponding point on the flattened cortex, $a$ reflects the proportion of V1 devoted to the foveal representation, $k$ is an overall scaling factor, and *squish* represents a scaling factor for the y (imaginary) dimension on the cortex. For most simulations we used standard parameters of *a* = *0.5*, *k* = *15*, and *squish* = 1.

To estimate the predicted locations of electrodes for Beauchamp et al.^25^ we simulated the implanted eCoG electrode array (4 × 6 configuration, 0.25mm radius mm electrodes, 2mm separation). We used function minimization to find the cortical shape (*a* = *0.15, k* = *16.6, squish* = *0.63*) and array position (*x* = *− 68.4, y* = *− 6.85,* and *angle* = *-2.2*) that best predicted the location of all 24 perceived phosphenes. These parameters fell within typical variation observed in human cortical maps^106^.

#### Orientation columns and ocular dominance maps

Based on Rojer and Schwarz^43^, orientation ‘pinwheel’ maps (Panel B) were simulated by filtering a 2-D complex-valued white noise image with an isotropic (unoriented) bandpass radial Gabor filter:

$$\text{F}=\frac{1}{2\uppi {{\sigma }_{F}}^{2}}{e}^{-\frac{1}{2}\left(\frac{{{x}^{2}+y}^{2}}{{{\sigma }_{F}}^{2}}\right)}cos\left({\omega }_{F}\sqrt{{x}^{2}+{y}^{2}}\right)$$

The angle of the resulting complex-valued image was used as the preferred orientation.

Ocular dominance columns were then simulated by calculating the gradient of that same filtered complex image in the x (real) dimension. This gradient image was then passed through a cumulative normal function to translate the gradient values ranging from below to above zero to an ocular dominance map with values ranging from zero to one. The result is an ocular dominance map whose columns overlap with the orientation map in a manner consistent with results from optical imaging data from Obermayer and Blasdel^41^.

Based on Adams et al.^42^ and similar to previously reported values^41^, ${\upomega }_{F}$, the millimeters per mean dominance column period, was set to 0.863. The width of the Gabor, ${\upsigma }_{F}$, which controls the spatial frequency range of the ocular dominance columns was set to 3 cycles/mm (a very narrow filter results in sinusoidal ocular dominance columns, a very broad filter would result in the absence of columnar structure).

#### Receptive fields

We assumed that receptive field size, $\upsigma$, linearly increases with eccentricity with an intercept of 0.16 and a slope of 0.08, based on electrophysiological estimates of macaque neuronal receptive fields^44^. These are the smallest values reported in the literature. We also carried out simulations using receptive field estimates based on a meta-analysis of ten older physiological data sets^67^.

V1 receptive fields were modeled as the combination of two Gaussians. The on-subunit (ON) was modeled as a 2D Gaussian region with a long axis of ${\sigma }_{rf}$ and a short axis of ${\sigma }_{rf}/4$ that responds to bright stimuli and the off-subunit (OFF) was modeled as an identically sized Gaussian region that responds to dark stimuli. In the original paper by Mata and Ringach^45^ on- and off- subunits also contained regions suppressed by bright and dark stimuli respectively. We assumed that the phosphenes elicited by electrical stimulation reflected only the excitatory components of the receptive fields.

Separations (${\delta }_{on/off},$ normalized by receptive field area) between the on and off subunits of each receptive field were drawn from an exponential distribution, such that small separations were common and large separations were rare, with the rate of fall-off designed to match neurophysiological data^45^. The same 2-D complex-valued white noise image as were used to generate ocular dominance and orientation columns^46^ was bandpass filtered with a radial Gabor of frequency 2 ${\upomega }_{F}=1.726.$ The angle, *u*, of the resulting complex-valued image (which had a flat distribution) was converted to distance between the on and off subunits:

$${\delta }_{on/off} =A \cdot sign\left(u\right)\frac{-\text{log}\left(\left|u\right|/\pi \right)}{{\delta }_{on/off}}$$

where *A* is the area of the ellipse described by the long (${\sigma }_{rf})$ and short axis (${\sigma }_{rf}/4$) of the receptive field. Receptive fields were shifted spatially along the direction of the short axis.

The relative strength of the on- and off-subunits were simulated by calculating the gradient of that same filtered complex image in the x (real) dimension. This gradient image was passed through a cumulative normal function to translate the gradient values ranging from below to above zero to an w_on/off_ map with values ranging from zero to one. Finally, we assumed that the contribution of on subunits was weighted more heavily than the contribution of off subunits, $\omega =0.8$ enabling us to capture the phenomenon that phosphene brightness increases as a function of current.

$$cell response={w}_{on/off}\cdot ON+{\omega \cdot (1-w}_{on/off})OFF$$

Thus, each orientation pinwheel and ocular dominance column contained pinwheels smoothly transitioning between complex cells (${\delta }_{on/off} <{\sigma }_{rf}/2,$ overlapping on-and off-subunits) and simple cells (${\delta }_{on/off} >{\sigma }_{rf}/2,$ largely non-overlapping on and off subunits) and columns that transitioned smoothly between on-cells and off-cells.

#### Predicted phosphenes

We simulated predicted phosphenes over time as the linear sum of receptive field profiles (normalized by their area) at each cortical location, scaled by the stimulation intensity at that location at each moment in time.

Simulated phosphenes were represented as $\text{X}\times Y$ pixel grayscale images, where $\text{x}\in \left[1,X\right]$ and $\text{y}\in \left[1,Y\right]$, in visual co-ordinates. We used two methods to estimate phosphene area and shape.

When comparing estimates to patient drawings (Fig. 5), phosphenes were quantified based on image moments after having thresholded the simulated phosphene based on a drawing threshold, ${\uptheta }_{draw }=1$, to create a binarized image, $\text{I}\left(x,y\right)$. The best-fitting ellipse was estimated based on this binary image using image moments, ${M}_{ij}$, calculated as:

$${M}_{ij}={\sum }_{x}{\sum }_{y}{x}^{i}{y}^{j}I\left(x,y\right).$$

For simulations of electrode size as a function of eccentricity (Fig. 7) we estimated size by finding the standard deviation of the best-fitting 2D Gaussian. The advantage of this approach is that it avoided using an arbitrary ‘drawing threshold’ and was more robust to fitting percepts generated by very small electrodes that were irregular in shape.

#### Simulating optimal cortical sampling

We define *optimal cortical sampling* as the spacing of cortical electrodes that separates visual phosphenes by σ, the standard deviation of the phosphene. Optimal cortical sampling depends on the mapping function from visual space to visual cortex and the size of phosphenes as a function of visual eccentricity.

Paradoxically, for realistic phosphene sizes and a feasible map between visual space and cortex, optimal cortical sampling should be *less* dense toward the foveal representation of the visual field, despite the large expansion of cortex devoted to foveal vision.

Mathematically, this can be understood by considering the 1-dimensional case of projecting eccentricity, *x*, as a logarithmic function along the horizontal meridian onto cortical space *y*, where

$$y\left(x\right)= k\cdot log\left(x+a\right).$$

Let *σ(x)* be the function describing the size of the phosphene as a function of eccentricity, *x*. This phosphene will span the range from $x-\frac{\upsigma (\text{x})}{2}\: \text{to} \: x+\frac{\upsigma (\text{x})}{2}$ along the horizontal meridian. The phosphene’s projection onto the cortex will have size:

$$\uprho \left(\text{x}\right)=\text{y}\left(x+\frac{\upsigma \left(\text{x}\right)}{2}\right)-\text{y}\left(x-\frac{\upsigma \left(\text{x}\right)}{2}\right).$$

This can be considered the optimal spacing between electrodes on the cortex, since any two electrodes with spacing less than $\uprho(x)$ will have overlapping phosphenes.

The first order Taylor expansion of *y(x)* allows the approximations:

$$\text{y}\left(x+\frac{\upsigma \left(\text{x}\right)}{2}\right) \sim y\left(x\right)+\frac{\upsigma \left(\text{x}\right)}{2}{y}^{\prime}\left(x\right).$$

And

$$\text{y}\left(x-\frac{\upsigma \left(\text{x}\right)}{2}\right) \sim y\left(x\right) - \frac{\upsigma \left(\text{x}\right)}{2}{y}^{\prime}\left(x\right).$$

So

$$\uprho \left(x\right) \sim\upsigma \left(x\right){y}^{\prime}\left(x\right).$$

Thus, the optimal spacing on the cortex is approximately equal to the size of the phosphene in visual space multiplied by the slope of the cortical mapping function *y(x)*. For our mapping function of $y=k\cdot log\left(x+a\right)$, ${y}^{\prime}\left(x\right)=\frac{k}{x+a}$, so

$$\uprho \left(x\right) \sim\upsigma \left(x\right)\frac{k}{x+a}.$$

We next assume that phosphene size grows as a linear function with eccentricity, $\upsigma \left(x\right)=mx+b$.

Substituting this into the equation above, and expressing eccentricity, x, as a function of cortical position, y, by inverting the mapping function, $x = {{\text{e}}^{\frac{{\text{y}}}{{\text{k}}}}} - {\text{a}}$, it follows that the optimal sampling on the cortex is:

$$s \left( {y} \right) = \frac{{{k} \cdot {m}\left( {{{e}^{\frac{{y}}{{k}}} + {a}}} \right) + {k} \cdot {b}}}{{{{e}^{\frac{{y}}{{k}}}}}}.$$

At the fovea, *x* = *0*, *y* = *log(a)*, and $s = \frac{k\cdot b}{a}$ In the far periphery, $s$ asymptotes to $s = k\cdot m$. If $m=\frac{b}{a}$ then the optimal sampling would be constant across the cortex. If $m<\frac{b}{a}$ then the optimal electrode spacing on the cortical surface in the fovea is greater than in the periphery. We assumed cortical mapping parameters of *k* = *15* mm and *a* = *0.5* deg in our simulations, based on cortical maps from Refs.^39,106^. We assumed an intercept of *b* = *0.16* and a slope of *m* = *0.08*, based on electrophysiological estimates of macaque neuronal receptive fields^44^. For these values, the optimal sampling, $s$(y), is 2.2 mm at 1 degree eccentricity and decreases to 1.3 mm at 20 degrees eccentricity.

#### Quantification and statistical analysis

Our model was designed to qualitatively rather than quantitatively replicate the predicted effects of cortical stimulation.

### Supplementary Information

Supplementary Video 1.

Supplementary Video 2.

Supplementary Video 3.

Supplementary Video 4.

Supplementary Video 5.

Supplementary Video 6.

Supplementary Information.

Supplementary Figure 1.

Supplementary Figure 2.

Supplementary Figure 3.

Supplementary Figure 4.

## Data Availability

Summary data values used for modeling are included in the github repository containing the model (https://github.com/VisCog/p2p-cortical).

## References

[1] Wood, E. H. *et al.* Stem cell therapies, gene-based therapies, optogenetics, and retinal prosthetics: Current state and implications for the future. *Retina (Philadelphia, Pa)* **39**, 820 (2019). 30664120 10.1097/IAE.0000000000002449 PMC6492547

[2] Rizzo, S. *et al.* The argus II retinal prosthesis: 12-month outcomes from a single-study center. *Am. J. Ophthalmol.* **157**, 1282–1290. 10.1016/j.ajo.2014.02.039 (2014). 24560994 10.1016/j.ajo.2014.02.039

[3] da Cruz, L. *et al.* Five-year safety and performance results from the argus II retinal prosthesis system clinical trial. *Ophthalmology* **123**, 2248–2254. 10.1016/j.ophtha.2016.06.049 (2016). 27453256 10.1016/j.ophtha.2016.06.049 PMC5035591

[4] Stingl, K. *et al.* Subretinal visual implant alpha IMS–clinical trial interim report. *Vis. Res.* **111**, 149–160. 10.1016/j.visres.2015.03.001 (2015). 25812924 10.1016/j.visres.2015.03.001

[5] Hornig, R. *et al.**Artificial Vision: A Clinical Guide* 99–113 (Springer International Publishing, 2017).

[6] Lorach, H. *et al.* Photovoltaic restoration of sight with high visual acuity. *Nat. Med.* **21**, 476–482. 10.1038/nm.3851 (2015). 25915832 10.1038/nm.3851 PMC4601644

[7] Ayton, L. N. *et al.* First-in-human trial of a novel suprachoroidal retinal prosthesis. *PLoS One* **9**, 1–26. 10.1371/journal.pone.0115239 (2014). 10.1371/journal.pone.0115239 PMC4270734 25521292

[8] Saunders, A. L. *et al.* Development of a surgical procedure for implantation of a prototype suprachoroidal retinal prosthesis. *Clin. Exp. Ophthalmol.* **42**, 665–674. 10.1111/ceo.12287 (2014). 24330322 10.1111/ceo.12287 PMC4233968

[9] Fujikado, T. *et al.* One-year outcome of 49-channel suprachoroidal-transretinal stimulation prosthesis in patients with advanced retinitis pigmentosa. *Investig. Ophthalmol. Vis. Sci.* **57**, 6147–6157. 10.1167/iovs.16-20367 (2016). 27835711 10.1167/iovs.16-20367

[10] Palanker, D., Le Mer, Y., Mohand-Said, S. & Sahel, J. A. Simultaneous perception of prosthetic and natural vision in AMD patients. *Nat. Commun.* **13**, 513. 10.1038/s41467-022-28125-x (2022). 35082313 10.1038/s41467-022-28125-x PMC8792035

[11] Simunovic, M. *et al.* Optogenetic approaches to vision restoration. *Exp. Eye Res.* **178**, 15–26 (2019). 30218651 10.1016/j.exer.2018.09.003

[12] McClements, M. E., Staurenghi, F., MacLaren, R. E. & Cehajic-Kapetanovic, J. Optogenetic gene therapy for the degenerate retina: recent advances. *Front. Neurosci.* **14**, 570909 (2020). 33262683 10.3389/fnins.2020.570909 PMC7686539

[13] Sahel, J.-A. *et al.* Partial recovery of visual function in a blind patient after optogenetic therapy. *Nat. Med.* **27**, 1223–1229 (2021). 34031601 10.1038/s41591-021-01351-4

[14] AbbVie. RST-001 Phase I/​II trial for advanced retinitis pigmentosa (NCT02556736) (2022).

[15] Bionic Sight LLC. BS01 in patients with retinitis pigmentosa (NCT04278131) (2023).

[16] Varela, M. D., de Guimaraes, T. A. C., Georgiou, M. & Michaelides, M. Leber congenital amaurosis/early-onset severe retinal dystrophy: Current management and clinical trials. *Br. J. Ophthalmol.* **106**, 445–451 (2022). 33712480 10.1136/bjophthalmol-2020-318483 PMC8961750

[17] Varela, M. D., Georgiadis, T. & Michaelides, M. Genetic treatment for autosomal dominant inherited retinal dystrophies: Approaches, challenges and targeted genotypes. *Br. J. Ophthalmol.* **107**, 1223–1230 (2022). 36038193 10.1136/bjo-2022-321903

[18] Kashani, A. H. Stem cell-derived retinal pigment epithelium transplantation in age-related macular degeneration: recent advances and challenges. *Curr. Opin. Ophthalmol.* **33**, 211–218 (2022). 35200164 10.1097/ICU.0000000000000838

[19] Zarbin, M., Sugino, I. & Townes-Anderson, E. Concise review: Update on retinal pigment epithelium transplantation for age-related macular degeneration. *Stem Cells Transl. Med.* **8**, 466–477 (2019). 30748126 10.1002/sctm.18-0282 PMC6477002

[20] Shen, Y. Stem cell therapies for retinal diseases: From bench to bedside. *J. Mol. Med.* **98**, 1347–1368 (2020). 32794020 10.1007/s00109-020-01960-5

[21] John, M. C., Quinn, J., Hu, M. L., Cehajic-Kapetanovic, J. & Xue, K. Gene-agnostic therapeutic approaches for inherited retinal degenerations. *Front. Mol. Neurosci.*10.3389/fnmol.2022.1068185 (2023). 36710928 10.3389/fnmol.2022.1068185 PMC9881597

[22] Wang, H., Yang, Y., Liu, J. & Qian, L. Direct cell reprogramming: Approaches, mechanisms and progress. *Nat. Rev. Mol. Cell Biol.* **22**, 410–424 (2021). 33619373 10.1038/s41580-021-00335-z PMC8161510

[23] Antolik, J., Sabatier, Q., Galle, C., Frégnac, Y. & Benosman, R. Assessment of optogenetically-driven strategies for prosthetic restoration of cortical vision in large-scale neural simulation of V1. *Sci. Rep.* **11**, 10783 (2021). 34031442 10.1038/s41598-021-88960-8 PMC8144184

[24] Bosking, W. H. *et al.* Saturation in phosphene size with increasing current levels delivered to human visual cortex. *J. Neurosci.* **37**, 7188–7197. 10.1523/JNEUROSCI.2896-16.2017 (2017). 28652411 10.1523/JNEUROSCI.2896-16.2017 PMC5546398

[25] Beauchamp, M. S. *et al.* Dynamic stimulation of visual cortex produces form vision in sighted and blind humans. *Cell* **181**, 774-783e775. 10.1016/j.cell.2020.04.033 (2020). 32413298 10.1016/j.cell.2020.04.033 PMC7331799

[26] Troyk, P. R. *Artificial Vision* (Springer International Publishing, 2017).

[27] Fernández, E. *et al.* Visual percepts evoked with an intracortical 96-channel microelectrode array inserted in human occipital cortex. *J. Clin. Investig.*10.1172/jci151331 (2021). 34665780 10.1172/jci151331 PMC8631600

[28] Brindley, G. S. & Lewin, W. S. The sensations produced by electrical stimulation of the visual cortex. *J. Physiol.* **196**, 479–493 (1968). 4871047 10.1113/jphysiol.1968.sp008519 PMC1351724

[29] Dobelle, W. H. & Mladejovsky, M. G. Phosphenes produced by electrical stimulation of human occipital cortex, and their application to the development of a prosthesis for the blind. *J. Physiol.* **243**, 553–576 (1974). 4449074 10.1113/jphysiol.1974.sp010766 PMC1330721

[30] Rushton, D. & Brindley, G. Short-and long-term stability of cortical electrical phosphenes. In *Physiological Aspects of Clinical Neurology* (ed. Rose, F. C.) 123–153 (Blackwell, 1977).

[31] Evans, J. R., Gordon, J., Abramov, I., Mladejovsky, M. G. & Dobelle, W. H. Brightness of phosphenes elicited by electrical stimulation of human visual cortex. *Sens. Process.* **3**, 82–94 (1979). 515743

[32] Girvin, J. *et al.* Electrical stimulation of human visual cortex: the effect of stimulus parameters on phosphene threshold. *Sens. Process.* **3**, 66–81 (1979). 515742

[33] Dobelle, W. H., Quest, D. O., Antunes, J. L., Roberts, T. S. & Girvin, J. P. Artificial vision for the blind by electrical stimulation of the visual cortex. *Neurosurgery* **5**, 521–527 (1979). 534058 10.1227/00006123-197910000-00022

[34] Schmidt, E. M. *et al.* Feasibility of a visual prosthesis for the blind based on intracortical microstimulation of the visual cortex. *Brain* **119**, 507–522 (1996). 8800945 10.1093/brain/119.2.507

[35] Winawer, J. & Parvizi, J. Linking electrical stimulation of human primary visual cortex, size of affected cortical area, neuronal responses, and subjective experience. *Neuron* **92**, 1213–1219. 10.1016/j.neuron.2016.11.008 (2016). 27939584 10.1016/j.neuron.2016.11.008 PMC5182175

[36] Oswalt, D. *et al.* Multi-electrode stimulation evokes consistent spatial patterns of phosphenes and improves phosphene mapping in blind subjects. *Brain Stimul.* **14**, 1356–1372. 10.1016/j.brs.2021.08.024 (2021). 34482000 10.1016/j.brs.2021.08.024 PMC8488973

[37] Bosking, W. H. *et al.* Percepts evoked by multi-electrode stimulation of human visual cortex. *Brain Stimul.* **15**, 1163–1177. 10.1016/j.brs.2022.08.007 (2022). 35985472 10.1016/j.brs.2022.08.007 PMC9831085

[38] Salas, M. A. *et al.* Sequence of visual cortex stimulation affects phosphene brightness in blind subjects. *Brain Stimul.* **15**, 605–614. 10.1016/j.brs.2022.03.008 (2022). 35378336 10.1016/j.brs.2022.03.008

[39] Schwartz, E. L. A quantitative model of the functional architecture of human striate cortex with application to visual illusion and cortical texture analysis. *Biol. Cybern.* **37**, 63–76 (1980). 6772241 10.1007/BF00364246

[40] Schwartz, E. L. Computational anatomy and functional architecture of striate cortex: A spatial mapping approach to perceptual coding. *Vis. Res.* **20**, 645–669 (1980). 7445436 10.1016/0042-6989(80)90090-5

[41] Obermayer, K. & Blasdel, G. G. Geometry of orientation and ocular dominance columns in monkey striate cortex. *J. Neurosci.* **13**, 4114–4129 (1993). 8410181 10.1523/JNEUROSCI.13-10-04114.1993 PMC6576395

[42] Adams, D. L., Sincich, L. C. & Horton, J. C. Complete pattern of ocular dominance columns in human primary visual cortex. *J. Neurosci.* **27**, 10391–10403. 10.1523/JNEUROSCI.2923-07.2007 (2007). 17898211 10.1523/JNEUROSCI.2923-07.2007 PMC6673158

[43] Rojer, A. S. & Schwartz, E. L. Cat and monkey cortical columnar patterns modeled by bandpass-filtered 2D white noise. *Biol. Cybern.* **62**, 381–391 (1990). 2110005 10.1007/BF00197644

[44] Keliris, G. A., Li, Q., Papanikolaou, A., Logothetis, N. K. & Smirnakis, S. M. Estimating average single-neuron visual receptive field sizes by fMRI. *Proc. Natl. Acad. Sci. U. S. A.* **116**, 6425–6434. 10.1073/pnas.1809612116 (2019). 30867291 10.1073/pnas.1809612116 PMC6442598

[45] Mata, M. L. & Ringach, D. L. Spatial overlap of ON and OFF subregions and its relation to response modulation ratio in macaque primary visual cortex. *J. Neurophysiol.* **93**, 919–928. 10.1152/jn.00668.2004 (2005). 15371494 10.1152/jn.00668.2004

[46] Najafian, S. *et al.* A theory of cortical map formation in the visual brain. *Nat. Commun.* **13**, 2303. 10.1038/s41467-022-29433-y (2022). 35484133 10.1038/s41467-022-29433-y PMC9050665

[47] Greenwald, S. H. *et al.* Brightness as a function of current amplitude in human retinal electrical stimulation. *Investig. Ophthalmol. Vis. Sci.* **50**, 5017–5025. 10.1167/iovs.08-2897 (2009). 19608533 10.1167/iovs.08-2897 PMC2798064

[48] Beyeler, M. *et al.* A model of ganglion axon pathways accounts for percepts elicited by retinal implants. *Sci. Rep.* **9**, 9199. 10.1038/s41598-019-45416-4 (2019). 31235711 10.1038/s41598-019-45416-4 PMC6591412

[49] Tehovnik, E. J., Tolias, A. S., Sultan, F., Slocum, W. M. & Logothetis, N. K. Direct and indirect activation of cortical neurons by electrical microstimulation. *J. Neurophysiol.* **96**, 512–521. 10.1152/jn.00126.2006 (2006). 16835359 10.1152/jn.00126.2006

[50] Ringach, D. L. Spatial structure and symmetry of simple-cell receptive fields in macaque primary visual cortex. *J. Neurophysiol.* **88**, 455–463. 10.1152/jn.2002.88.1.455 (2002). 12091567 10.1152/jn.2002.88.1.455

[51] Rahimi-Nasrabadi, H. *et al.* Image luminance changes contrast sensitivity in visual cortex. *Cell Rep.* **34**, 108692. 10.1016/j.celrep.2021.108692 (2021). 33535047 10.1016/j.celrep.2021.108692 PMC7886026

[52] Kremkow, J. *et al.* Neuronal nonlinearity explains greater visual spatial resolution for darks than lights. *Proc. Natl. Acad. Sci. U. S. A.* **111**, 3170–3175. 10.1073/pnas.1310442111 (2014). 24516130 10.1073/pnas.1310442111 PMC3939872

[53] Pons, C. *et al.* Neuronal mechanisms underlying differences in spatial resolution between darks and lights in human vision. *J. Vis.* **17**, 5. 10.1167/17.14.5 (2017). 29196762 10.1167/17.14.5 PMC5713488

[54] Nowak, L. G. & Bullier, J. Axons, but not cell bodies, are activated by electrical stimulation in cortical gray matter. I. Evidence from chronaxie measurements. *Exp. Brain Res.* **118**, 477–488. 10.1007/s002210050304 (1998). 9504843 10.1007/s002210050304

[55] Lapicque, L. Quantitative investigations of electrical nerve excitation treated as polarization 1907. *Biol. Cybern.* **97**, 341–349. 10.1007/s00422-007-0189-6 (2007). 18046573 10.1007/s00422-007-0189-6

[56] Knight, B. W. Dynamics of encoding in a population of neurons. *J. Gen. Physiol.* **59**, 734–766. 10.1085/jgp.59.6.734 (1972). 5025748 10.1085/jgp.59.6.734 PMC2203203

[57] Fellner, A., Heshmat, A., Werginz, P. & Rattay, F. A finite element method framework to model extracellular neural stimulation. *J. Neural Eng.* **19**, 022001 (2022). 10.1088/1741-2552/ac6060 35320783

[58] Rattay, F. *Electrical Nerve Stimulation* (Springer, 1990).

[59] Rattay, F., Paredes, L. P. & Leao, R. N. Strength-duration relationship for intra- versus extracellular stimulation with microelectrodes. *Neuroscience* **214**, 1–13. 10.1016/j.neuroscience.2012.04.004 (2012). 22516015 10.1016/j.neuroscience.2012.04.004 PMC3401985

[60] Rattay, F. Analysis of models for external stimulation of axons. *IEEE Trans. Biomed. Eng.*10.1109/TBME.1986.325670 (1986). 3770787 10.1109/TBME.1986.325670

[61] Jensen, R. J., Rizzo, J. F. III., Ziv, O. R., Grumet, A. & Wyatt, J. Thresholds for activation of rabbit retinal ganglion cells with an ultrafine, extracellular microelectrode. *Investig. Ophthalmol. Vis. Sci.* **44**, 3533–3543 (2003). 12882804 10.1167/iovs.02-1041

[62] Jensen, R. J., Ziv, O. R. & Rizzo, J. F. 3rd. Thresholds for activation of rabbit retinal ganglion cells with relatively large, extracellular microelectrodes. *Investig. Ophthalmol. Vis. Sci.* **46**, 1486–1496 (2005). 15790920 10.1167/iovs.04-1018

[63] Polimeni, J. R., Balasubramanian, M. & Schwartz, E. L. Multi-area visuotopic map complexes in macaque striate and extra-striate cortex. *Vis. Res* **46**, 3336–3359. 10.1016/j.visres.2006.03.006 (2006). 16831455 10.1016/j.visres.2006.03.006 PMC2248457

[64] Schwartz, E. L. *Cerebral Cortex* (Plenum Press, 1994).

[65] Benson, N. C. *et al.* The retinotopic organization of striate cortex is well predicted by surface topology. *Curr. Biol.* **22**, 2081–2085. 10.1016/j.cub.2012.09.014 (2012). 23041195 10.1016/j.cub.2012.09.014 PMC3494819

[66] Yacoub, E., Harel, N. & Ugurbil, K. High-field fMRI unveils orientation columns in humans. *Proc. Natl. Acad. Sci. U. S. A.* **105**, 10607–10612. 10.1073/pnas.0804110105 (2008). 18641121 10.1073/pnas.0804110105 PMC2492463

[67] Freeman, J. & Simoncelli, E. P. Metamers of the ventral stream. *Nat. Neurosci.* **14**, 1195–1201. 10.1038/nn.2889 (2011). 21841776 10.1038/nn.2889 PMC3164938

[68] Tehovnik, E. J. & Slocum, W. M. Phosphene induction by microstimulation of macaque V1. *Brain Res. Rev.* **53**, 337–343. 10.1016/j.brainresrev.2006.11.001 (2007). 17173976 10.1016/j.brainresrev.2006.11.001 PMC1850969

[69] Lapicque, L. *L’excitabilité en Fonction du Temps: La Chronaxie, sa Signification et sa Mesure* Vol. 4 (Les presses universitaires de France, 1926).

[70] Horsager, A. *et al.* Predicting visual sensitivity in retinal prosthesis patients. *Investig. Ophthalmol. Vis. Sci.* **50**, 1483–1491. 10.1167/iovs.08-2595 (2009). 19098313 10.1167/iovs.08-2595 PMC2729061

[71] Bosking, W. H., Beauchamp, M. S. & Yoshor, D. Electrical stimulation of visual cortex: Relevance for the development of visual cortical prosthetics. *Annu. Rev. Vis. Sci.* **3**, 141–166. 10.1146/annurev-vision-111815-114525 (2017). 28753382 10.1146/annurev-vision-111815-114525 PMC6916716

[72] Hinds, O. P. *et al.* Accurate prediction of V1 location from cortical folds in a surface coordinate system. *Neuroimage* **39**, 1585–1599. 10.1016/j.neuroimage.2007.10.033 (2008). 18055222 10.1016/j.neuroimage.2007.10.033 PMC2258215

[73] Allman, J. M. & Kaas, J. H. The organization of the second visual area (V II) in the owl monkey: A second order transformation of the visual hemifield. *Brain Res.* **76**, 247–265. 10.1016/0006-8993(74)90458-2 (1974). 4210762 10.1016/0006-8993(74)90458-2

[74] Van Essen, D. C., Newsome, W. T. & Maunsell, J. H. The visual field representation in striate cortex of the macaque monkey: Asymmetries, anisotropies, and individual variability. *Vis. Res.* **24**, 429–448. 10.1016/0042-6989(84)90041-5 (1984). 6740964 10.1016/0042-6989(84)90041-5

[75] Gattass, R., Gross, C. G. & Sandell, J. H. Visual topography of V2 in the macaque. *J. Comp. Neurol.* **201**, 519–539. 10.1002/cne.902010405 (1981). 7287933 10.1002/cne.902010405

[76] Gattass, R., Sousa, A. P. & Rosa, M. G. Visual topography of V1 in the Cebus monkey. *J. Comp. Neurol.* **259**, 529–548. 10.1002/cne.902590404 (1987). 3597827 10.1002/cne.902590404

[77] Cavanaugh, J. R., Bair, W. & Movshon, J. A. Nature and interaction of signals from the receptive field center and surround in macaque V1 neurons. *J. Neurophysiol.* **88**, 2530–2546. 10.1152/jn.00692.2001 (2002). 12424292 10.1152/jn.00692.2001

[78] Cavonius, C. R. & Robbins, D. O. Relationships between luminance and visual acuity in the rhesus monkey. *J. Physiol.* **232**, 239–246. 10.1113/jphysiol.1973.sp010267 (1973). 4199366 10.1113/jphysiol.1973.sp010267 PMC1350452

[79] Ridder, W. H., Zhang, K. M., Karsolia, A., Engles, M. & Burke, J. Comparison of contrast sensitivity in macaque monkeys and humans. *Vis. Neurosci.* **36**, E008. 10.1017/s0952523819000051 (2019). 31199217 10.1017/s0952523819000051

[80] Curcio, C. A., Sloan, K. R., Kalina, R. E. & Hendrickson, A. E. Human photoreceptor topography. *J. Comp. Neurol.* **292**, 497–523. 10.1002/cne.902920402 (1990). 2324310 10.1002/cne.902920402

[81] Daniel, P. & Whitteridge, D. The representation of the visual field on the cerebral cortex in monkeys. *J. Physiol.* **159**, 203 (1961). 13883391 10.1113/jphysiol.1961.sp006803 PMC1359500

[82] Stevens, C. F. Predicting functional properties of visual cortex from an evolutionary scaling law. *Neuron* **36**, 139–142. 10.1016/s0896-6273(02)00902-9 (2002). 12367512 10.1016/s0896-6273(02)00902-9

[83] Grinten, M. V. D. *et al.* Biologically plausible phosphene simulation for the differentiable optimization of visual cortical prostheses. *bioRxiv*10.1101/2022.12.23.521749 (2022). 10.1101/2022.12.23.521749

[84] Dobelle, W. H. Artificial vision for the blind by connecting a television camera to the visual cortex. *ASAIO J.* **46**, 3–9 (2000). 10667705 10.1097/00002480-200001000-00002

[85] Troyk, P. et al. In *2015 7th International IEEE/EMBS Conference on Neural Engineering (NER)*, 474–477 (IEEE).

[86] Barry, M. P. *et al.* Contributed session III: Characteristics of electrically-induced visual percepts in the first human with the intracortical visual prosthesis. *J. Vis.* **23**, 35–35. 10.1167/jov.23.11.35 (2023). 10.1167/jov.23.11.35

[87] Rubinstein, J. T., Spelman, F. A., Soma, M. & Suesserman, M. F. Current density profiles of surface mounted and recessed electrodes for neural prostheses. *IEEE Trans. Biomed. Eng.* **34**, 864–875 (1987). 3319885 10.1109/TBME.1987.326007

[88] Suesserman, M. F., Spelman, F. A. & Rubinstein, J. T. In vitro measurement and characterization of current density profiles produced by non-recessed, simple recessed, and radially varying recessed stimulating electrodes. *IEEE Trans Biomed. Eng.* **38**, 401–408 (1991). 1874521 10.1109/10.81558

[89] Cone, J. J., Ni, A. M., Ghose, K. & Maunsell, J. H. R. Electrical microstimulation of visual cerebral cortex elevates psychophysical detection thresholds. *eNeuro*10.1523/eneuro.0311-18.2018 (2018). 30406199 10.1523/eneuro.0311-18.2018 PMC6220593

[90] Horsager, A., Greenberg, R. J. & Fine, I. Spatiotemporal interactions in retinal prosthesis subjects. *Investig. Ophthalmol. Vis. Sci.* **51**, 1223–1233. 10.1167/iovs.09-3746 (2010). 19741248 10.1167/iovs.09-3746 PMC2868442

[91] Brackbill, N. *et al.* Reconstruction of natural images from responses of primate retinal ganglion cells. *eLife* **9**, e58516. 10.7554/eLife.58516 (2020). 33146609 10.7554/eLife.58516 PMC7752138

[92] Benson, N. C., Butt, O. H., Brainard, D. H. & Aguirre, G. K. Correction of distortion in flattened representations of the cortical surface allows prediction of v1–v3 functional organization from anatomy. *PLoS Comput. Biol.* **10**, e1003538. 10.1371/journal.pcbi.1003538 (2014). 24676149 10.1371/journal.pcbi.1003538 PMC3967932

[93] Von Helmholtz, H. (Voss Hamburg, 1909).

[94] Westheimer, G. Editorial: Visual acuity and hyperacuity. *Investig. Ophthalmol.* **14**, 570–572 (1975). 1150397

[95] Gogliettino, A. R. *et al.* High-fidelity reproduction of visual signals by electrical stimulation in the central primate retina. *J. Neurosci.* **43**, 4625–4641. 10.1523/jneurosci.1091-22.2023 (2023). 37188516 10.1523/jneurosci.1091-22.2023 PMC10286946

[96] Fine, I. & Boynton, G. M. Pulse trains to percepts: The challenge of creating a perceptually intelligible world with sight recovery technologies. *Philos. Trans. R. Soc. B Biol. Sci.* **370**, 20140208 (2015). 10.1098/rstb.2014.0208 PMC4528820 26240423

[97] Encke, J., Benav, H., Werginz, P., Zrenner, E. & Rattay, F. Investigating the influence of 3D cell morphology on neural response during electrical stimulation. *Biomed. Tech. Biomed. Eng.*10.1515/bmt-2013-4035 (2013). 10.1515/bmt-2013-4035 24042619

[98] Werginz, P., Benav, H., Encke, J., Zrenner, E. & Rattay, F. Neural activation for different electrode designs in subretinal implants: A modeling study. *Biomed. Tech. Biomed. Eng.*10.1515/bmt-2013-4036 (2013). 10.1515/bmt-2013-4036 24042606

[99] Rattay, F. & Resatz, S. Effective electrode configuration for selective stimulation with inner eye prostheses. *IEEE Trans. Biomed. Eng.* **51**, 1659–1664. 10.1109/tbme.2004.828044 (2004). 15376514 10.1109/tbme.2004.828044

[100] Dagnelie, G., Barnett, D., Humayun, M. S. & Thompson, R. W. Jr. Paragraph text reading using a pixelized prosthetic vision simulator: Parameter dependence and task learning in free-viewing conditions. *Investig. Ophthalmol. Vis. Sci.* **47**, 1241–1250. 10.1167/iovs.05-0157 (2006). 16505065 10.1167/iovs.05-0157

[101] Thompson, R. W. Jr., Barnett, G. D., Humayun, M. S. & Dagnelie, G. Facial recognition using simulated prosthetic pixelized vision. *Investig. Ophthalmol. Vis. Sci.* **44**, 5035–5042. 10.1167/iovs.03-0341 (2003). 14578432 10.1167/iovs.03-0341

[102] Esquenazi, R. B., Meier, K., Beyeler, M., Boynton, G. M. & Fine, I. Learning to see again: Perceptual learning of simulated abnormal on- off-cell population responses in sighted individuals. *J. Vis.* **21**, 10. 10.1167/jov.21.13.10 (2021). 34935878 10.1167/jov.21.13.10 PMC8727313

[103] Kasowski, J. & Beyeler, M. Immersive virtual reality simulations of bionic vision. *Augment. Hum.* **2022**(2022), 82–93. 10.1145/3519391.3522752 (2022). 10.1145/3519391.3522752 PMC9289996 35856703

[104] Granley, J., Fauvel, T., Chalk, M. & Beyeler, M. Human-in-the-loop optimization for deep stimulus encoding in visual prostheses. Preprint https://arXiv.org//2306.13104 (2023). PMC11232484 38984104

[105] Nanduri, D. *et al.* Frequency and amplitude modulation have different effects on the percepts elicited by retinal stimulation. *Investig. Ophthalmol. Vis. Sci.* **53**, 205–214. 10.1167/iovs.11-8401 (2012). 22110084 10.1167/iovs.11-8401 PMC3292357

[106] Duncan, R. O. & Boynton, G. M. Cortical magnification within human primary visual cortex correlates with acuity thresholds. *Neuron* **38**, 659–671 (2003). 12765616 10.1016/S0896-6273(03)00265-4

