# Supplementary material for: A virtual patient simulation modeling the neural and perceptual effects of human visual cortical stimulation, from pulse trains to percepts
Source: Sci Rep. 2024 Jul 29;14:17400. doi: 10.1038/s41598-024-65337-1 (PMC11286872; doi:10.1038/s41598-024-65337-1)
Supplement: Supplementary file 7 — Supplementary Information. [file 41598_2024_65337_MOESM7_ESM.pdf]

SUPPLEMENTAL MATERIALS

Supplemental Figure 1. Phosphene brightness (upper panel) and size as a function of stimulation frequency and amplitude. Simulationswere carried out at 2.5 deg eccentricity using an electrode of 0.5 mm radius

Supplemental Videos for Figure 6.

Beauchamp et al.[1] stimulated selected electrodes to generate four different "letter" percepts. Successive electrodes in each trajectory were stimulated with small amounts of current (~1 mA) at high frequency (~200 Hz) either simultaneously or in rapid temporal sequence (50 ms per electrode, 50 ms delay between each electrode).

To estimate the predicted locations of electrodes for Beauchamp et al.[1] we simulated the implanted eCoG electrode array (4 x 6 configuration, 0.25mm radius mm electrodes, 2mm separation). We used function minimization to find the cortical shape ($a = 0.15, k = 16.6, squish = 0.63$) and array position ($x = -68.4, y = -6.85,$ and $angle = -2.2$) that best predicted the location of all 24 perceived phosphenes.

LettersPatientLocation.mov
Contains:

- C Simultaneous Stimulation, Patient Locations for phosphenes
- C Sequential Stimulation, Patient Locations for phosphenes
- N Simultaneous Stimulation, Patient Locations for phosphenes
- N Sequential Stimulation, Patient Locations for phosphenes
- S Simultaneous Stimulation, Patient Locations for phosphenes
- S Sequential Stimulation, Patient Locations for phosphenes
- U Simultaneous Stimulation, Patient Locations for phosphenes
- U Sequential Stimulation, Patient Locations for phosphenes

LettersEstimatedLocation.mov
Contains:

- C Simultaneous Stimulation, Estimated Locations for phosphenes based on a grid
- C Sequential Stimulation, Estimated Locations for phosphenes based on a grid
- N Simultaneous Stimulation, Estimated Locations for phosphenes based on a grid
- N Sequential Stimulation, Estimated Locations for phosphenes based on a grid
- S Simultaneous Stimulation, Estimated Locations for phosphenes based on a grid
- S Sequential Stimulation, Estimated Locations for phosphenes based on a grid
- U Simultaneous Stimulation, Estimated Locations for phosphenes based on a grid
- U Sequential Stimulation, Estimated Locations for phosphenes based on a grid

Supplemental Figures 2-4 and Videos for Figure 8.

Simulations comparing different electrode array configurations.

Supplemental Figures 2-4 show arrays with decreasing electrode density. The left panel shows the electrode placement on the cortical surface of V1, the middle panel shows simulated phosphenes as a function of eccentricity. (A) Regular spacing of electrodes on the visual field. (B)

Regular spacing of electrodes on the cortical surface. (C) 'Optimal' spacing (the center-to-center separation of phosphenes is a constant proportion of phosphene size).

ArraySimulations_Spacing_1.mov
Simulations with 1884-2540 electrodes
Contains:

- Array with Optimal spacing with large (.25mm) and point electrodes
- Array with Regular Spacing on cortex with large (.25mm) and point electrodes
- Array with Electrodes Spaced to create phosphenes that are regular in the visual field with large (0.25mm) and point electrodes

ArraySimulations_Spacing_2.mov
Simulations with 399-590 electrodes
Contains:

- Array with Optimal spacing with large (.25mm) and point electrodes
- Array with Regular Spacing on cortex with large (.25mm) and point electrodes
- Array with Electrodes Spaced to create phosphenes that are regular in the visual field with large (0.25mm) and point electrodes

ArraySimulations_Spacing_3.mov
Simulations with 163-232 electrodes
Contains:

- Array with Optimal spacing with large (.25mm) and point electrodes
- Array with Regular Spacing on cortex with large (.25mm) and point electrodes
- Array with Electrodes Spaced to create phosphenes that are regular in the visual field with large (0.25mm) and point electrodes

ArraySimulations_Spacing_4.mov
Simulations with 79-120 electrodes
Contains:

- Array with Optimal spacing with large (.25mm) and point (0.001mm) electrodes
- Array with Regular Spacing on cortex with large (.25mm) and point (0.001mm) electrodes
- Array with Electrodes Spaced to create phosphenes that are regular in the visual field with large (0.25mm) and point (0.001mm) electrodes

References

1. Beauchamp, M.S., Oswalt, D., Sun, P., Foster, B.L., Magnotti, J.F., Niketeghad, S., Pouratian, N., Bosking, W.H., and Yoshor, D. (2020). Dynamic Stimulation of Visual Cortex Produces Form Vision in Sighted and Blind Humans. Cell *181*, 774-783 e775. 10.1016/j.cell.2020.04.033.
