# Supplementary figures and images for: A virtual patient simulation modeling the neural and perceptual effects of human visual cortical stimulation, from pulse trains to percepts
Source: Sci Rep. 2024 Jul 29;14:17400. doi: 10.1038/s41598-024-65337-1 (PMC11286872; doi:10.1038/s41598-024-65337-1)

Supplementary Figure 1

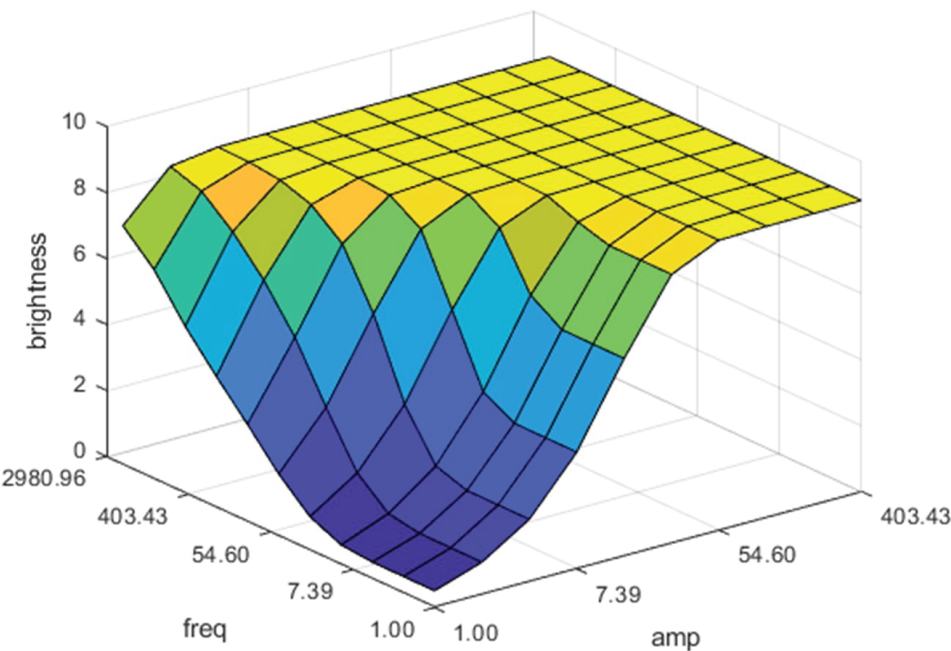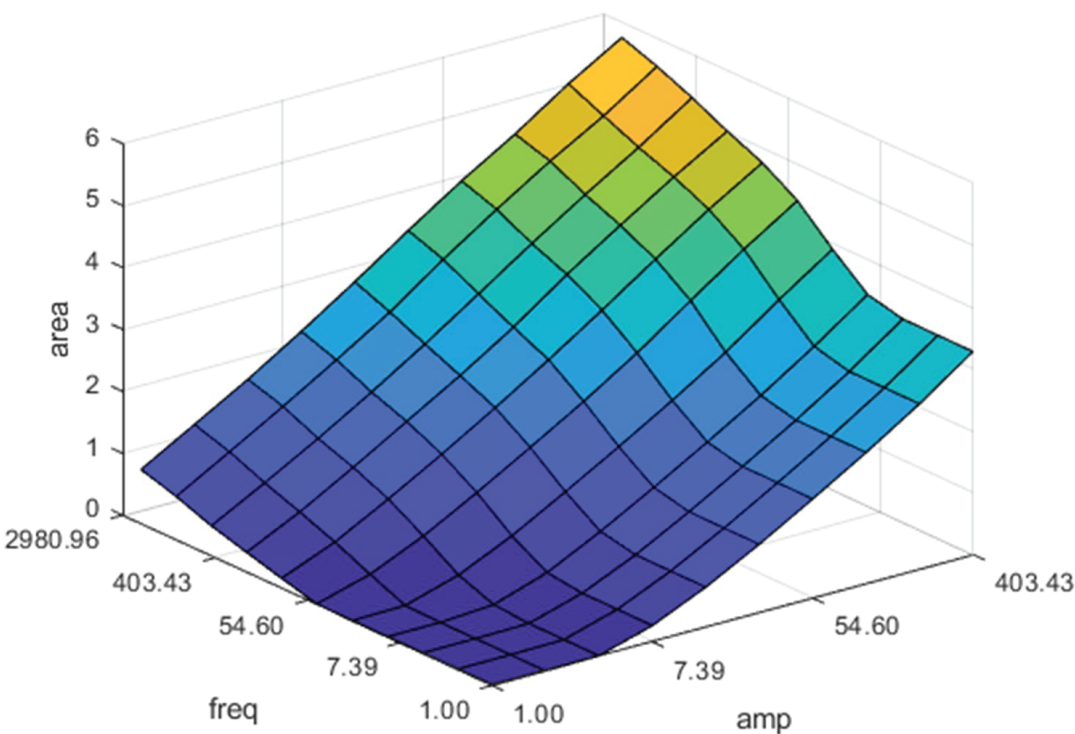

Supplement: Supplementary file 8 — Supplementary Figure 1. [file 41598_2024_65337_MOESM8_ESM.pdf]

## Supplementary Figure 2

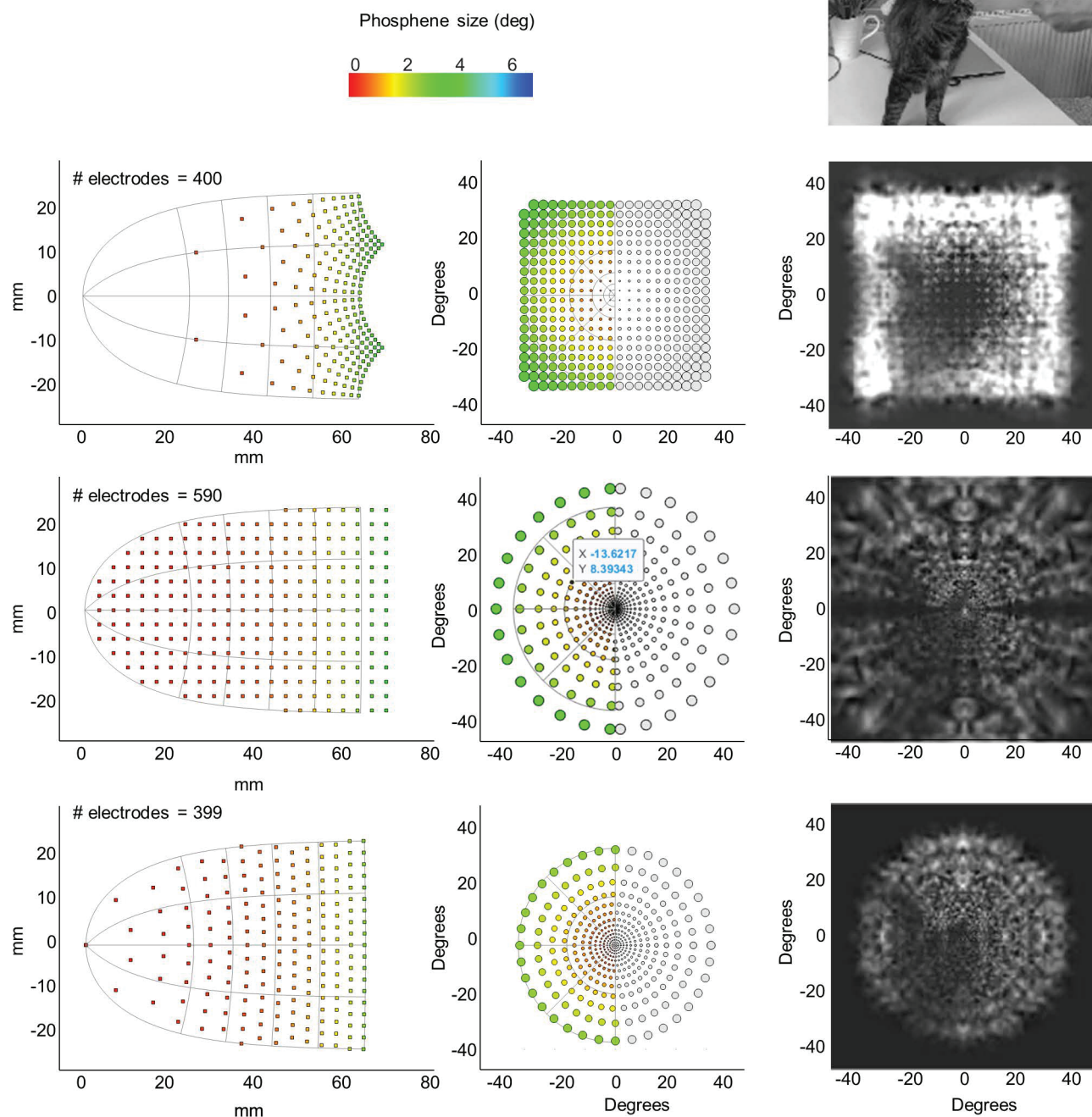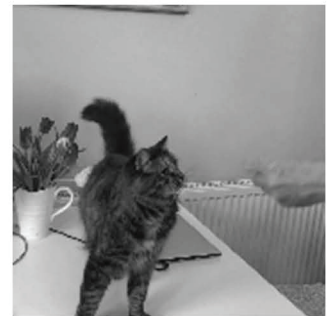

Supplement: Supplementary file 9 — Supplementary Figure 2. [file 41598_2024_65337_MOESM9_ESM.pdf]

Supplementary Figure 3

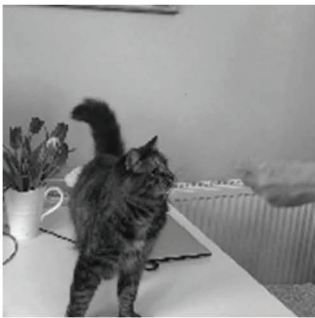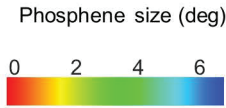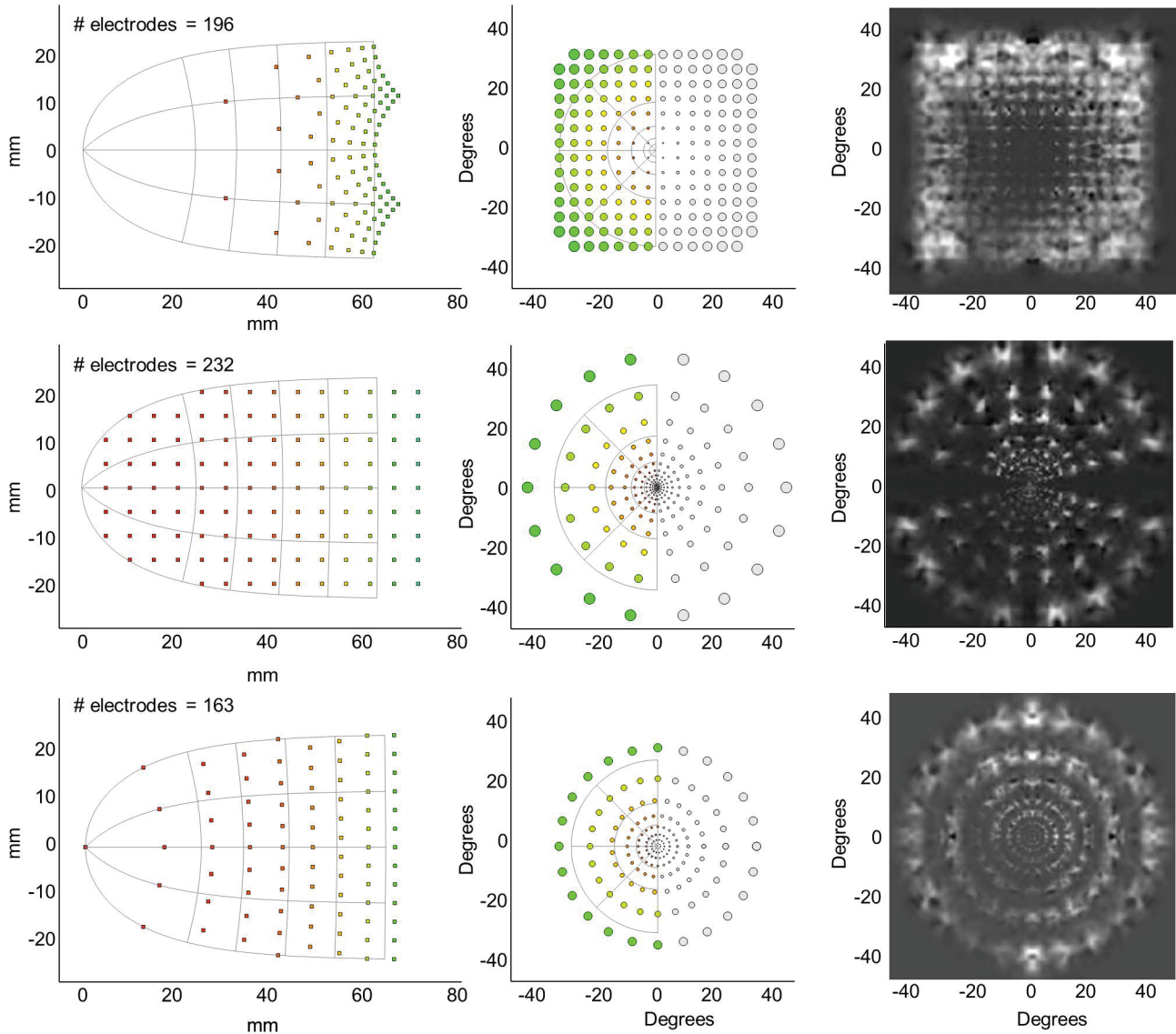

Supplement: Supplementary file 10 — Supplementary Figure 3. [file 41598_2024_65337_MOESM10_ESM.pdf]

Supplementary Figure 4

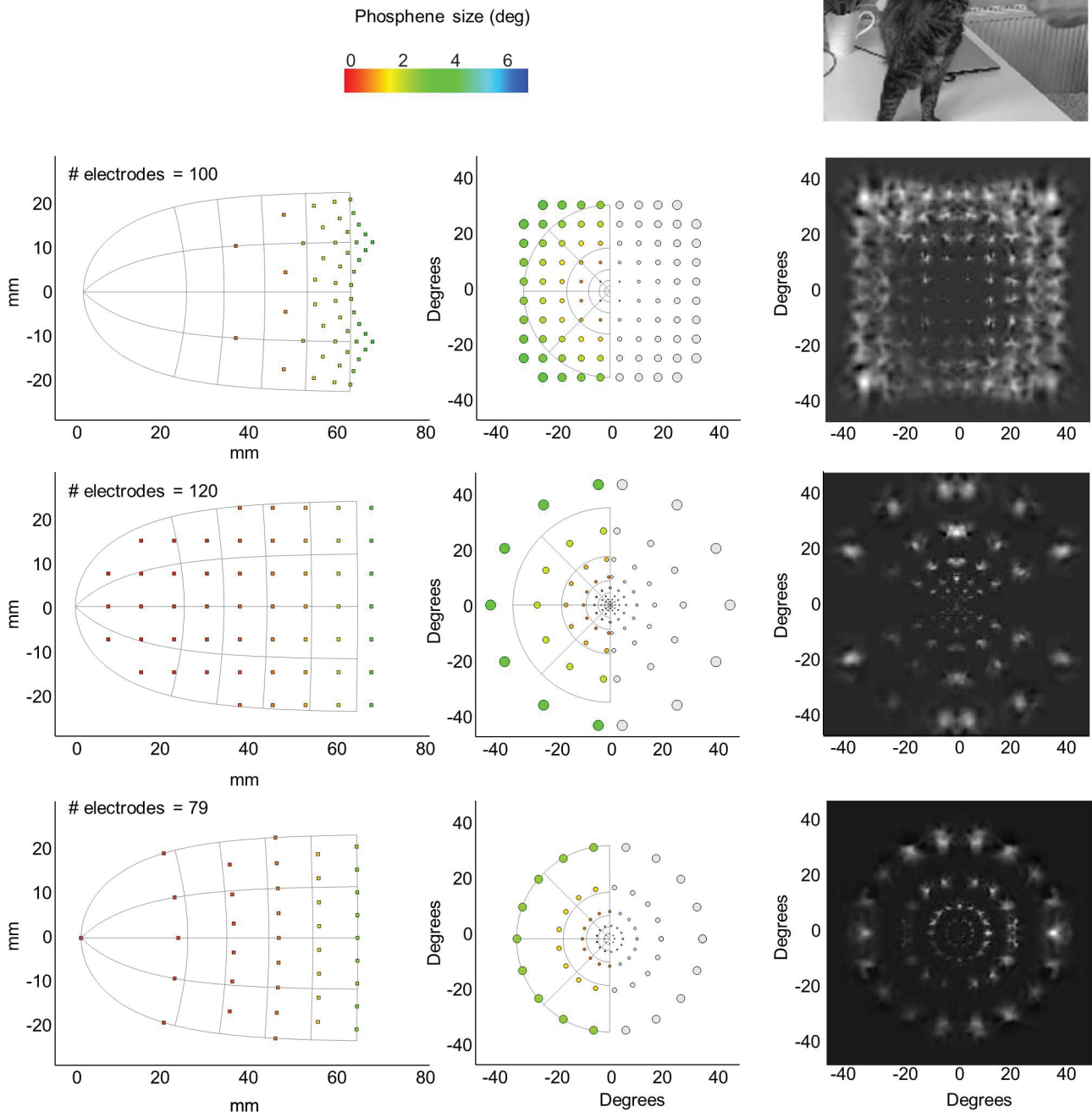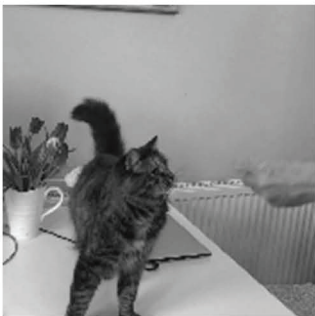

Supplement: Supplementary file 11 — Supplementary Figure 4. [file 41598_2024_65337_MOESM11_ESM.pdf]
